# Comparative Study of Molecular Mechanics Force Fields for β-Peptidic Foldamers: Folding and Self-Association

**DOI:** 10.1021/acs.jcim.3c00175

**Published:** 2023-06-06

**Authors:** András Wacha, Zoltán Varga, Tamás Beke-Somfai

**Affiliations:** Institute of Materials and Environmental Chemistry, Research Centre for Natural Sciences, Loránd Eötvös Research Network, Magyar tudósok körútja 2, H-1117 Budapest, Hungary

## Abstract

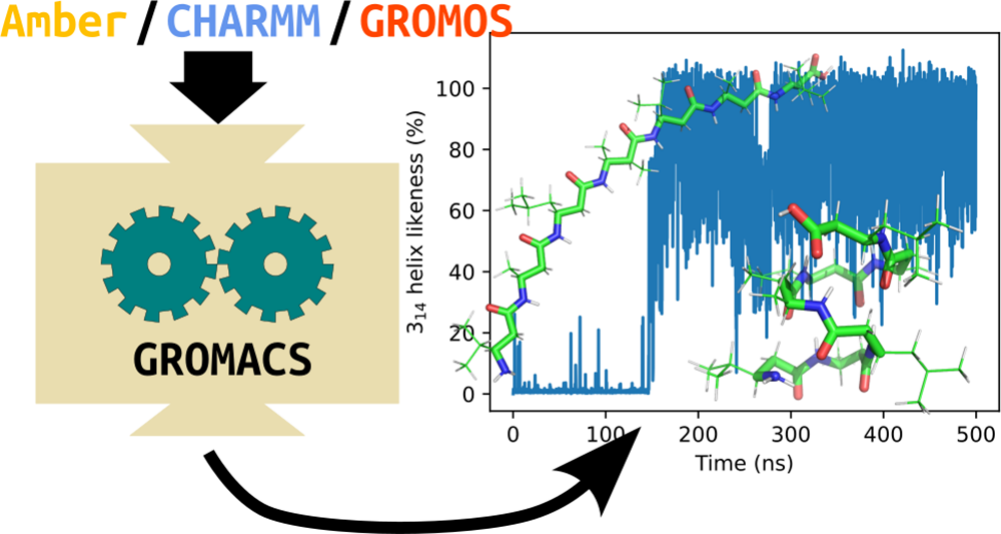

Computer-assisted study and design of non-natural peptidomimetics is increasingly important in the development of novel constructs with widespread applicability. Among these methods, molecular dynamics can accurately describe monomeric as well as oligomeric states of these compounds. We studied seven different sequences composed of cyclic and acyclic β-amino acids, the closest homologues of natural peptides, and compared the performance on them of three force field families in which specific modifications were made to improve reproduction of β-peptide structures. Altogether 17 systems were simulated, each for 500 ns, testing multiple starting conformations and in three cases also oligomer formation and stability from eight β-peptide monomers. The results indicated that our recently developed CHARMM force field extension, based on torsional energy path matching of the β-peptide backbone against quantum-chemical calculations, performs best overall, reproducing the experimental structures accurately in all monomeric simulations and correctly describing all the oligomeric examples. The Amber and GROMOS force fields could only treat some of the seven peptides (four in each case) without further parametrization. Amber was able to reproduce the experimental secondary structure of those β-peptides which contained cyclic β-amino acids, while the GROMOS force field had the lowest performance in this sense. From the latter two, Amber was able to hold together already formed associates in the prepared state but was not able to yield spontaneous oligomer formation in the simulations.

## 1 Introduction

Non-natural peptidic compounds have shown in the last two decades both great structural diversity and widespread applicability.^1−8^ One of the most extensively studied set of molecules among these are β-peptides, for which structural examples include helical structures,^4,6,9−12^ sheet-like conformations,^13^ hairpins,^10,14,15^ and even higher ordered aqueous and membrane-associated bundle oligomers^1,16−24^ and nanofibers.^25−27^ These molecules have now widespread potential applications in nanotechnology,^28−30^ biomedical fields,^4,31−37^ biopolymer surface recognition,^38−40^ catalysis,^16,41,42^ and biotechnology.^43−45^ Furthermore, the more complex, oligomeric bundles of helical foldamers constructed by Schepartz et al.^19^ show also potential in diverse fields, with demonstrated metal ion binding,^1^ catalytic,^16^ or carbohydrate sensing capacity.^46,47^

These examples strongly suggest that besides reaching functions similar to those of their natural counterparts, design of new peptidic assemblies may also give the opportunity to reach functions so far unseen for natural biomolecules. This is further suggested by some important structural differences between natural peptides and peptidic foldamers, primarily arising from the properties of the amino acid backbone. In the design of such novel systems, molecular simulations, especially molecular dynamics (MD) simulations, are indispensable, as these can provide principal molecular-level insight into the properties of the designed compounds and their assemblies even before commencing synthetic procedures. However, to this end the appropriate reproduction of their basic properties is crucial, as employing nonaccurate parameters can lead to erroneous conformational preferences or artifacts derailing the following experimental steps of a design procedure.

Various force fields (FFs), i.e., empirical interaction potential functions and the corresponding numeric parameters, have been developed over the time for the computational study of biomacromolecules, such as proteins, nucleic acids, carbohydrates, and other smaller molecules. Many of these have been extended for the case of β-peptides. One of the first attempts was as early as 1997 by the van Gunsteren group, the original developers of the GROMOS FF and molecular simulation engine.^48^ Up to this day, this is still the only FF that supports β-peptides “out of the box”. With the Amber FF family, two separate attempts are found in the literature: the work by the Gellman group, who presented the AMBER*C variant, validated for cyclic β-amino acids,^49^ and the work by the Martinek research group, who extended the FF for acyclic β-amino acids as well.^50^ Finally, the CHARMM FF was adapted by Cui and co-workers^51−53^ for this family of non-natural peptidomimetics.

In our previous work, we improved the attempt of the Cui group at extending the CHARMM force field to β-peptides by a rigorous study of backbone torsions.^54^ The elimination of correlations between dihedral angle parameters resulted in a better reconstruction of the *ab initio* potential energy surface and in a closer matching of the experimentally determined values of relevant structural quantities.

In the present work, we take the next step of comparing how the variants of three commonly used FFs (Amber, CHARMM, and GROMOS) tailored for β-peptides perform on a wide and representative selection of β-peptide sequences spanning various experimentally reported secondary structures as well as self-assembly/association behavior.

## 2 Methods

### 2.1 Peptide Sequences

**Figure 1:**
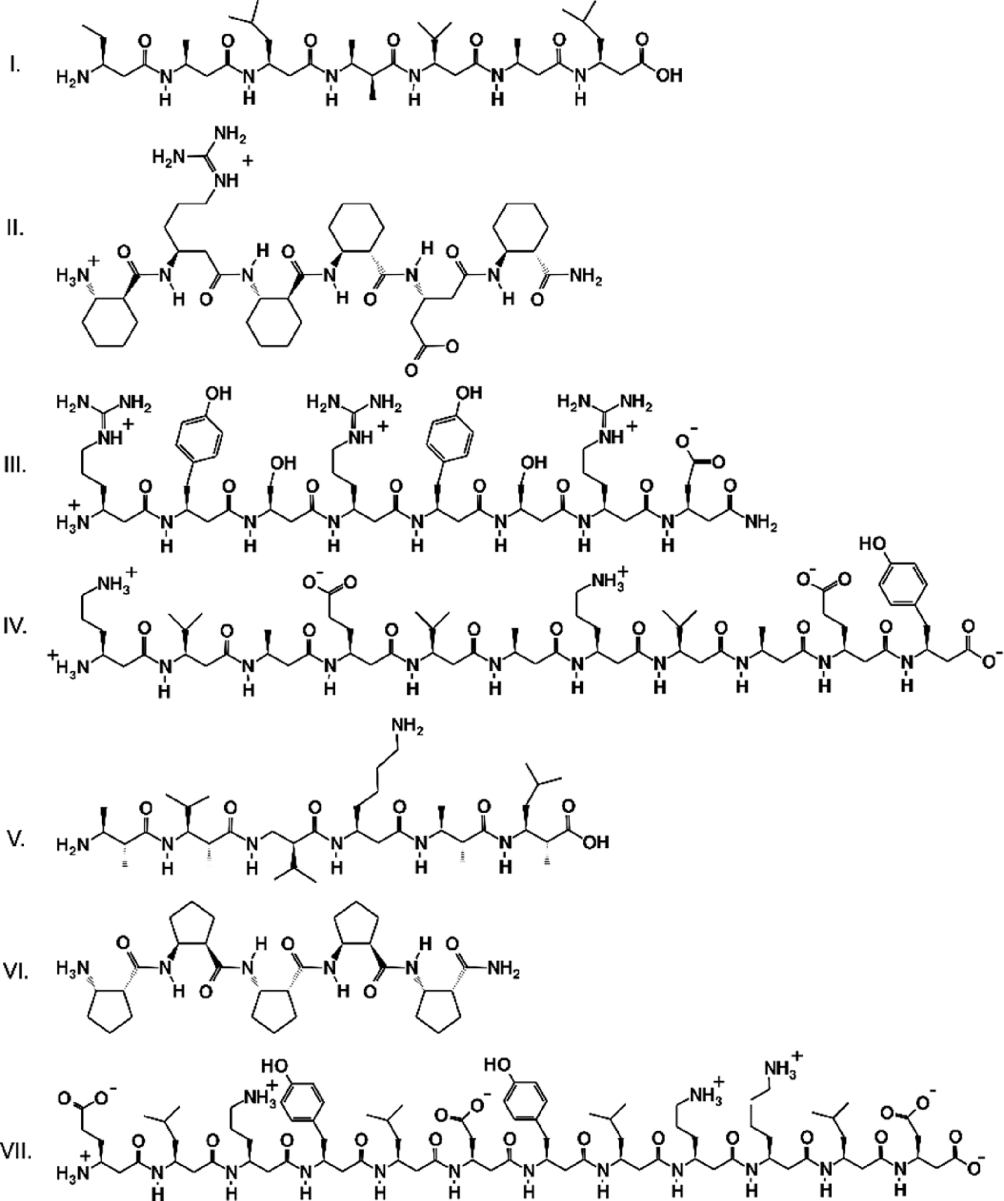
Studied peptide sequences.

Figure 1 lists the peptide sequences considered in the present work. Peptide I is a common benchmark for force fields. It has been reported to fold, when solvated in methanol, into a left-handed helix of 14-membered pseudorings and a full turn corresponding to approximately three β-amino acid residues. This 3_14_ structure has been extensively studied by both experimental methods (NMR) and MD.^48,53−57^ Peptides II, III, and VI were used as test cases by Németh et al. for their Amber-compatible molecular mechanics (MM) parameter derivation of cyclic and acyclic β-amino acids.^50^ Peptide II prefers the above-described 3_14_ helical conformation in aqueous media and was found to bind to synaptotoxic amyloid-β oligomers. In contrast, peptide III is disordered in water, without any long-range contact between residues.^50^ Finally, peptide VI was found to form isolated, elongated strands in dimethyl sulfoxide (DMSO)^58^ and assemble into nanostructured sheet-mimicking fibers in methanol and water.^59^ Peptide IV is among the first β-peptides composed exclusively of acyclic β-amino acids, which adopt a stable 3_14_ conformation in water, intended to act as inhibitors of protein–protein interactions.^60,61^

Peptide V represents a family of different secondary structures. It has been designed to adopt a hairpin-like conformation in aqueous solution.^14,15,54,62,63^ Our final example, peptide VII (denoted Zwit-EYYK by the original authors), has been designed to form stable octameric bundles in the shape of two cupped hands, each having four “fingers” of 3_14_ helices.^1,64^

### 2.2 Force Fields

All three employed force fields had explicit support for β-amino acids. The first one was based on the “official” GROMACS port^65^ of ff03,^66^ with new residue topologies constructed for β-amino acids upon analogy to their natural counterparts and partial charges refitted using the RESP method with "antechamber", as described by Németh et al.^50^ (details can be found in the Supporting Information). The second one was based on the March 2017 release of CHARMM36m force field, with dihedral angle potential energy parameters for the backbone derived from minimum energy path matching against *ab initio* calculations as reported by Wacha et al.^54^ Finally, two variants of the GROMOS force field were also tested, 54A7^67^ and 54A8,^68^ the former with the updates of Lin and van Gunsteren^69^ (data files dated May 2013 and November 2015, respectively). Both of these support β-amino acids out of the box, and therefore, no further modification was needed apart from deriving a β^3^-homoornithine residue, required by peptides IV and VII, by analogy to the already supported β^3^-homolysine (SBKH, protonated (*S*)-β3-homolysine).

### 2.3 Algorithms and Software

All three of the chosen force fields (Amber, CHARMM, and GROMOS) are closely tied to a corresponding molecular dynamics code of the same name. An impartial comparison requires that effects due to differences in the used algorithms and other details in their implementations be avoided, e.g., by using the same simulation engine. As none of the force-field-specific codes is able to fully handle the other two ones, we have chosen GROMACS as the common ground.^70−74^ Apart from being able to do simulations with all three force fields, this MD engine is known for its rigorously validated, physically sound, and highly parallelized algorithms, yielding an exceptional performance on modern hardware.^75,76^

MD simulations were performed using the 2019.5 version of GROMACS.^77^ For run preparation and trajectory analysis we have developed the “gmxbatch” Python package (to be published elsewhere).

Molecular models of the peptides were built using version 2.3.0 of the open source variant of the PyMOL molecular graphics system,^78,79^ employing “pmlbeta”, our extension for β-peptides.^80^

Molecular topologies for the Amber and CHARMM force field were generated using the "pdb2gmx" subprogram of GROMACS. For the GROMOS family of force fields, topologies (including interaction parameters) were created using the "make_top" and "OutGromacs" programs in the GROMOS software suite (version 1.4.1).

We should note here that in short peptides such as the ones in the present study, arbitrarily modifying the terminal groups can have a profound effect on the folding behavior. Therefore, special care was taken to perform all simulations with the correct termini applied as reported previously in the respective literature. From the three force fields, only CHARMM supported all required termini: Amber lacked neutral N- and C-termini, while GROMOS was missing the neutral amine and *N*-methylamide C-termini. The peptides which required these could not be simulated with the respective force fields.

After a short *in vacuo* energy minimization of a single chain with the steepest descent algorithm, the peptide molecules were folded by setting the backbone torsion angles to the values corresponding to the desired secondary structure of the initial state (folded or extended). Another energy minimization was done to remove residual strain, and then the peptide was placed in the center of a cubic box with at least a 1.4 nm peptide–wall distance. In those simulation runs where self-association was studied, a 0.5 nm peptide–box distance was used instead, and eight copies of the resulting box were assembled in a 2 × 2 × 2 cube after applying a random rotation to the peptide chain in each sub-box.

At this point, a box of pre-equilibrated solvent (water, methanol, or DMSO) was added, as well as neutralizing Na^+^ and Cl^–^ ions. For systems in water, additional salt was added in a concentration equivalent to 50 mM. Applying position restraints on the heavy atoms of the peptides, steepest descent energy minimization was performed on the solvent (including ions) to remove voids or steric clashes. Temperature coupling was turned on through a 100 ps MD run in the *NVT* ensemble using the weak coupling algorithm^81^ at 300 K temperature with τ = 1 ps coupling time. In order to avoid the hot solvent/cold solute problem, separate thermostats were used for the solvent and the peptide.^82^ Equilibration in the *NpT* ensemble was done during a 200 ps run with the same thermostat settings and an additional, isotropic barostat set at 1 bar with τ = 8 ps coupling time (20 ps for methanol to average out nonphysical oscillations occurring with the CHARMM force field) using the isothermal compressibility of water. In production runs, the velocity-rescaling thermostat with a stochastic extension was used,^83^ as the weak coupling algorithm does not sample the correct statistical ensemble. For the same reason, the Parrinello–Rahman barostat^84^ was used. Long-range electrostatic interactions were treated using the particle-mesh Ewald algorithm.^85^ Bond length constraints were handled by the LINCS algorithm in the case of Amber and CHARMM force fields.^86^ For the GROMOS force field, the SHAKE algorithm was used instead,^87^ partly to maintain compatibility with the GROMOS code and partly because LINCS, although faster and more stable, cannot handle coupled angle constraints and heavily linked molecules, such as the tetrahedral constraint graph of methanol.

At least two independent repeats were done for each case listed in Table 1, started from the same conformations but solvated and equilibrated separately. Trajectories of the full systems were recorded at 100 ps intervals. Additionally, reduced-precision “compressed” trajectories of the coordinates of everything but water were written every 10 ps, rounded to 3 decimal digits (expressed in nm units). Energy terms and various scalar parameters (density, box size, temperature, etc.) were also collected every 10 ps.

**Table 1:**
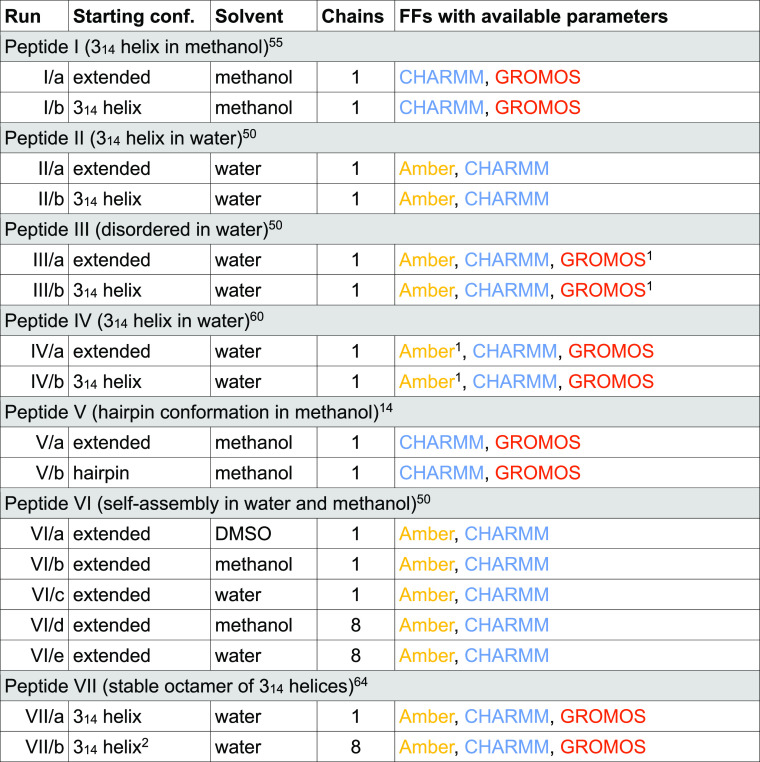
Summary of Simulations Performed

1 Simulations were carried out using different terminating groups.

2 The initial structure was taken from the Cambridge Crystallographic Database, deposition number 804687.^64^

### 2.4 Analysis of the Simulations

#### 2.4.1 Secondary Structure Characterization

As hydrogen bonding is the main stabilizing force behind peptide secondary structure, the folding/unfolding of the β-peptides can be conveniently followed by intrachain hydrogen bond fingerprinting.

A simple measure of the helical propensity of a β-peptide is the count of *i* → *i* + 2 intrachain hydrogen bonds. A continuous quantity can be derived based on the idea implemented in PLUMED.^88^ The presence or absence of intrachain hydrogen bonds is characterized by the switching function
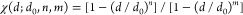
1where *d* is the distance between the hydrogen and the acceptor oxygen atom. With parameters *d*_0_ = 0.25 nm, *n* = 6, and *m* = 12, the function reaches 50% at 0.27 nm, while the 10% level corresponds to 0.44 nm. The measure of the helical propensity of a peptide consisting of *N* β-amino acids can then be defined as
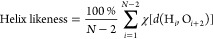
2where *i* enumerates the residues in the peptide of *N* amino acids and *d*(H_*i*_, O_*i*+2_) is the distance between the amide proton of residue *i* and the amide oxygen in residue *i* + 2. The largest possible number of hydrogen bonds (not counting multiples with terminal protons and oxygens) is therefore *N* – 2.

For the characterization of the conformational likeness to the hairpin structure of peptide V, a similar quantity can be employed by accounting for the presence of hydrogen bonds responsible and indicative for this folded structure:
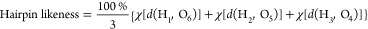
3

Another way to fingerprint the secondary structure of a peptide is through the residue–residue hydrogen bond occupancy map, showing the percentage of the total MD simulation time (including independent reruns of the same system) in which a pair of residues are linked by a hydrogen bond. Here the traditional binary, true-or-false approach based on distance and angle cutoffs,^89,90^ is better suited. We used the implementation by Smith et al.^91^ in the framework of the MDAnalysis library.^92,93^ The criteria of the presence of a hydrogen bond between the donor and the acceptor atoms are that the distance of the two atoms is less than 0.3 nm, the donor–hydrogen–acceptor angle is more than 150°, and the hydrogen is nearer to the donor than 0.12 nm.

#### 2.4.2 Characterization of Oligomeric Associates

Hydrogen bonds are also responsible for holding together oligomeric peptide associates. In our simulations, multichain associates are detected by an adapted version of the Hoshen–Kopelman cluster labeling algorithm,^94^ executed independently at each time step. The distinct peptide chains are sorted in equivalence classes based on pairwise connectivity via hydrogen bonds, detected by the discrete method of Smith et al. described above.

Through the trajectory, the birth times of dimers, trimers, tetramers, etc. are noted, and their lifetimes are followed while there exists a continuous network of however many hydrogen bonds joining them. Larger associates with more than two chains typically start and end their lives as dimers acquiring or losing a subsequent member chain. In our interpretation, the underlying smaller associate is also treated as “alive” while being part of a larger one.

#### 2.4.3 Agreement with Experimental Structures

Experimentally determined structural information may be used as basis for validating molecular trajectories obtained by computer simulation. Some of the peptides discussed in this work have structural data available, such as hydrogen–hydrogen distances determined through nuclear Overhauser effect spectroscopy (NOE) for peptides I, II, V, and VI in the respective literature. NOE violations were calculated for the distance between hydrogen atoms *i* and *j* as ⟨*r*_*ij*_^–6^(*t*)⟩^–1/6^ – *r*_*ij*_^exp^, where *r*_*ij*_ is the instantaneous distance between the atoms (or the smallest distance if there are more chemically equivalent atoms), *r*_*ij*_^exp^ is the reported NOE distance in the literature, and the averaging is with respect to simulation time. Due to the upper threshold nature, only positive violations needed to be considered.

For peptide VII, a crystal structure of the octameric bundle is available in the Cambridge Crystallographic Database. Structural divergence from this reference state was controlled by means of the root-mean-square deviation (RMSD) using the algorithm described by Theobald and implemented in MDAnlysis.^95^

## 3 Results and Discussion

### 3.1 Stability Analysis of 3_14_ Helices

As stated above, peptides I, II, IV, and VII are known to adopt a helical conformation with a 14-membered pseudoring in their respective solvents, while peptide III, although its sequence and the absolute conformation of the building blocks would permit it, prefers a random coil structure in water instead. A force field aiming to faithfully describe the folding properties of β-peptides should at least be able to keep such a conformation stable but desirably also allow and prefer spontaneous folding when started from a random configuration. The former property can be confirmed by simulations started from the appropriate helical conformation, while starting from a fully extended state should assess the performance of the FF in respect to the latter.

**Figure 2:**
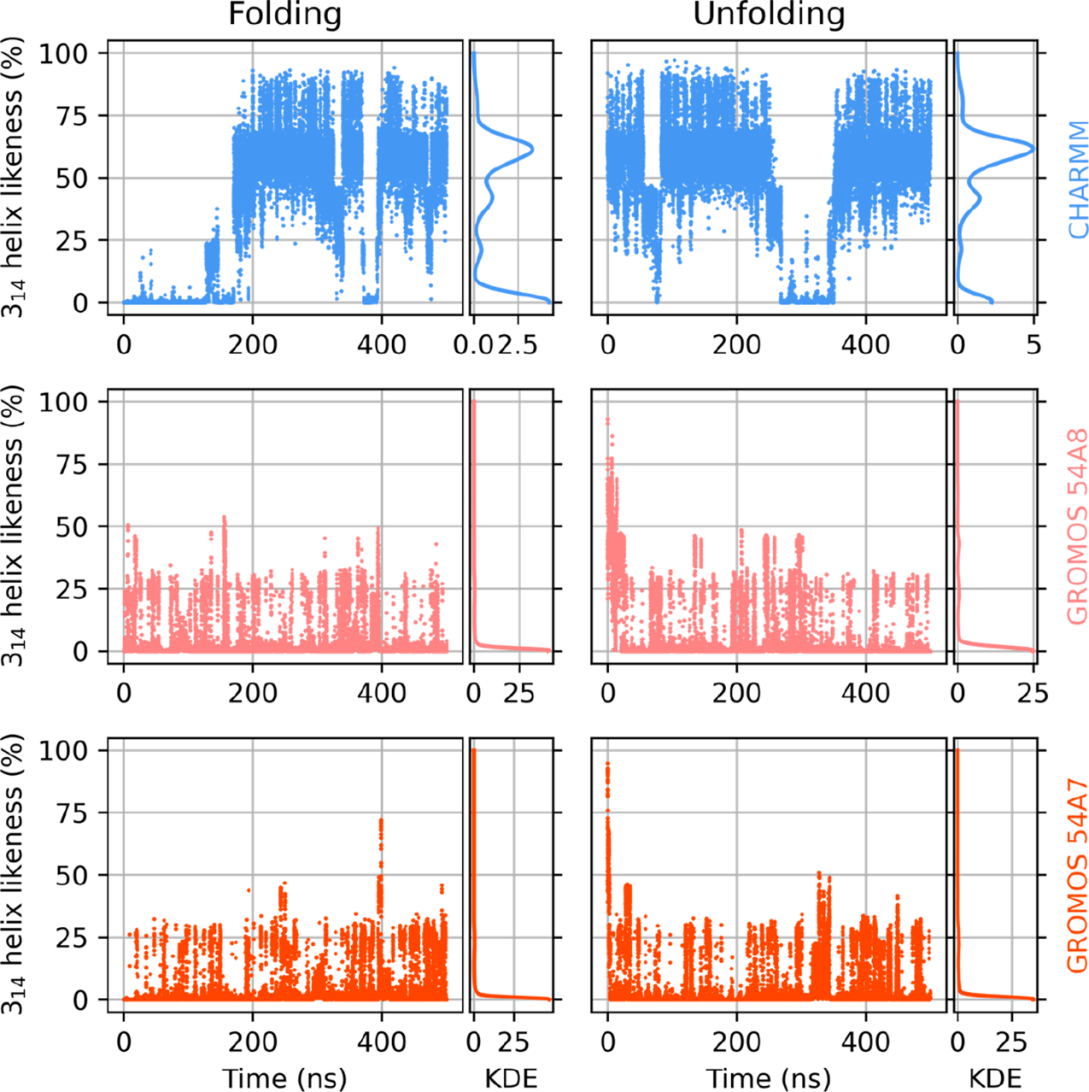
Evolution of the helicity of peptide I in methanol over time under various force fields: CHARMM (top row), GROMOS 54a8 (center row), and GROMOS 54A7 (bottom row), when started from extended (left column) and already helically folded (right column) conformations. Gaussian kernel density estimates are shown to the right of the respective time series.

The evolution of the helical propensity of peptide I in methanol during the MD trajectory (Figure 2), started from extended and helical conformations (simulations I/a and I/b in Table 1, respectively), can be calculated by eq 2. From the several independent runs performed, the best-case scenario with the highest overall helical propensity is shown for all FFs (all best- and worst-case time series are shown in Figures S18 and S19).

The CHARMM force can reproduce and keep the helical secondary structure, even allowing a temporary unfolding of the peptide, in line with the dynamic nature of β-peptide folding. This is most pronounced at the ends of the peptide chain, also causing the helix likeness score never to reach 100%.

Strangely, neither of the two variants of the GROMOS force field were able to recover the helix, in contrast to what the developers of the force field reported.^63,69^ The reason behind this may be that electrostatic and van der Waals interactions, the primary influencers of hydrogen bonding in MD simulations, have been parametrized in the GROMOS FF with a twin-range cutoff scheme, which, deemed not physically correct, is not supported by recent versions of the GROMACS code.^96^ Similar regression problems were found by several authors using GROMOS-compatible force fields with recent versions of the code.^97−100^

**Figure 3:**
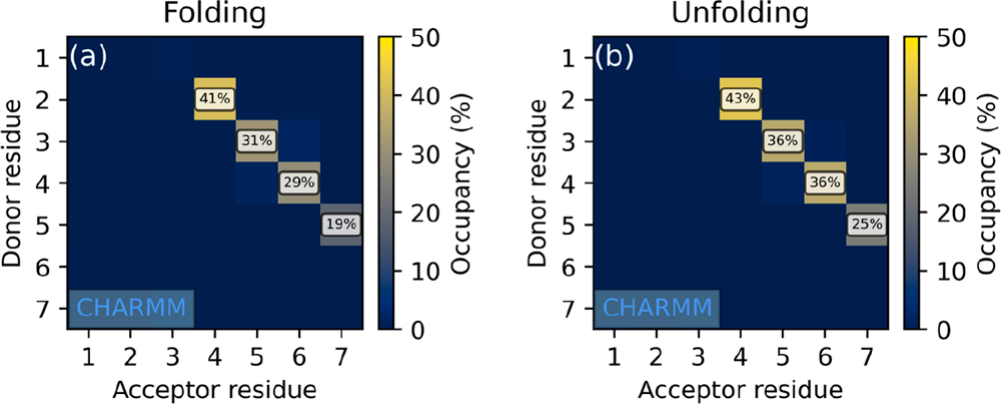
Hydrogen bond occupancy maps of peptide I simulated in methanol under the CHARMM FF, started from (a) extended and (b) 3_14_ helix conformations. Occupancies below 10% are not labeled.

The hydrogen bond occupancy maps (Figure 3) clearly show that the fingerprint of *i* → *i* + 2 hydrogen bonds characteristic for the 3_14_ helix is recovered by the CHARMM FF (similar figures including the two GROMOS variants can be found in Figures S1 and S2). The similarity of the two graphs confirms the independence of the sampled ensemble from the starting conformation. The looseness of the chain ends is also seen.

As the Amber force field has parameters only for charged termini, its performance on peptide I cannot be assessed due to the non-negligible chain-end effects incurred if the peptide would be differently terminated. Peptides II and III, however, are tractable with it, enabling the comparison of Amber and CHARMM.

Validation against experiments can be performed due to the availability of NOE results on peptide I, reported by Daura et al.^48^ Because GROMOS is a family of united atom force fields, aliphatic hydrogens are incorporated into their respective heavy atoms. While the positions of these could be reconstructed frame-by-frame, as is done in the GROMOS software,^101^ there was no point of doing this since the peptide is readily unfolded by both variants of this FF.

**Figure 4:**
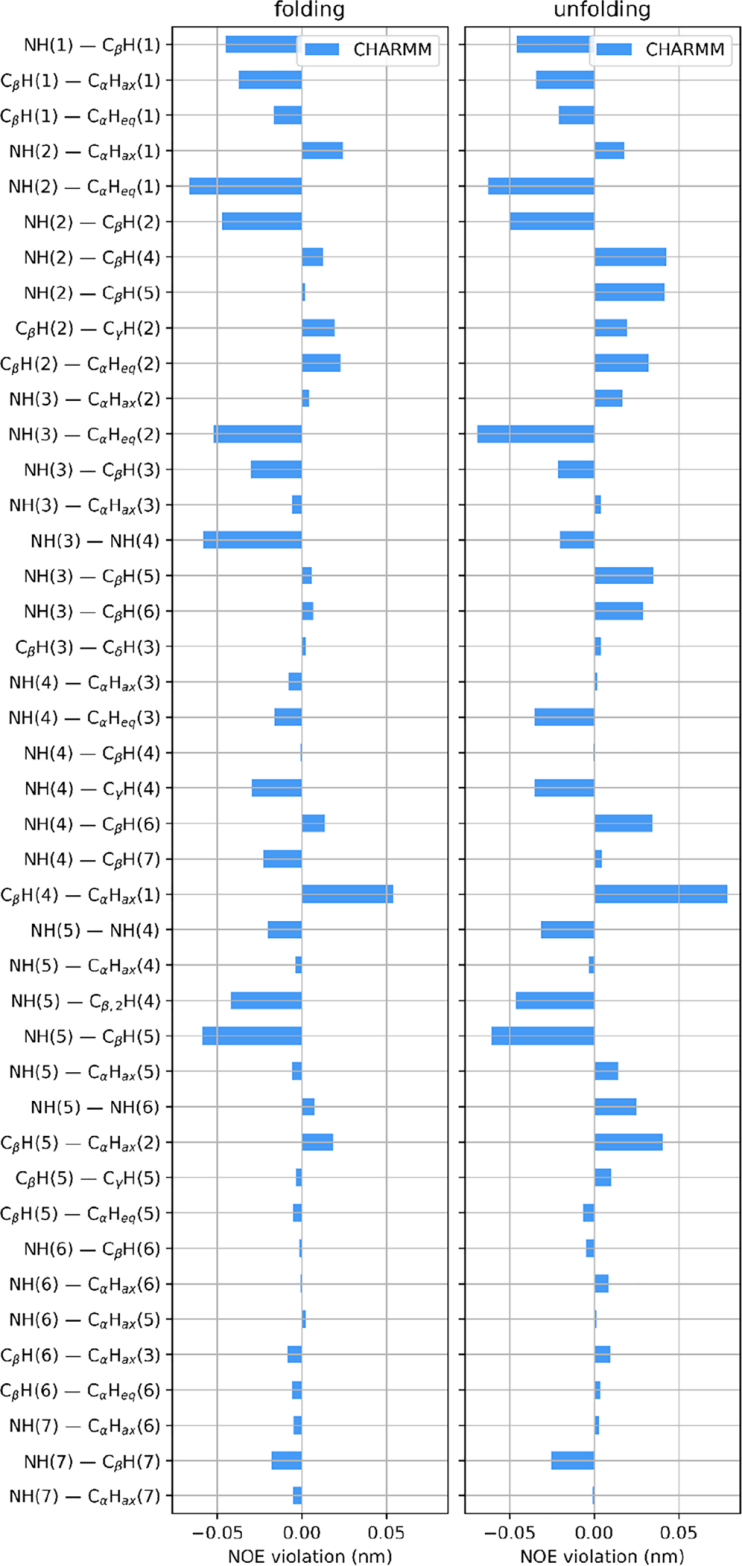
Effective violations of experimental hydrogen–hydrogen NOE distances for peptide I, started from (left) extended and (right) 3_14_ helical conformations. Averaging was done only on the last 300 ns of the trajectories.

The average NOE distance upper bound violations, determined from the last 300 ns of the trajectories (including independent reruns of the same system) are shown in Figure 4. Both folding directions show qualitatively the same behavior, with all the distance violations being less than 0.05 nm. The exception is the distance between the hydrogen of C_β_ of the fifth residue and the axial hydrogen of the C_α_ atom of the first N-terminal β-amino acid. The large violation is most possibly caused by the frequent unfolding/refolding at the termini due to the dynamic nature of the chain folding simulation.

**Figure 5:**
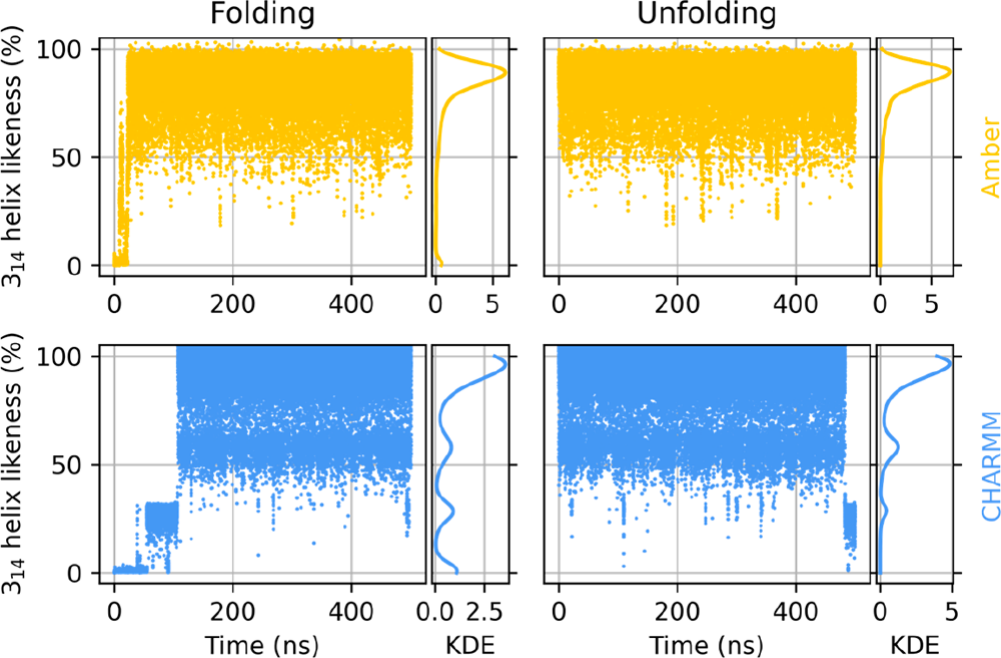
Evolution of the helicity of peptide II in water over time under Amber (top row) and CHARMM (bottom row) when started from extended (left column) and already helically folded (right column) conformations. Gaussian kernel density estimates are shown to the right of the respective time series.

Figure 5 shows the time evolution of the helicity measure of peptide II. Not unexpectedly, the 3_14_ helix conformation is readily obtained in a very short time (under 50 ns for Amber, slightly over 100 ns for CHARMM) and retained practically infinitely.

**Figure 6:**
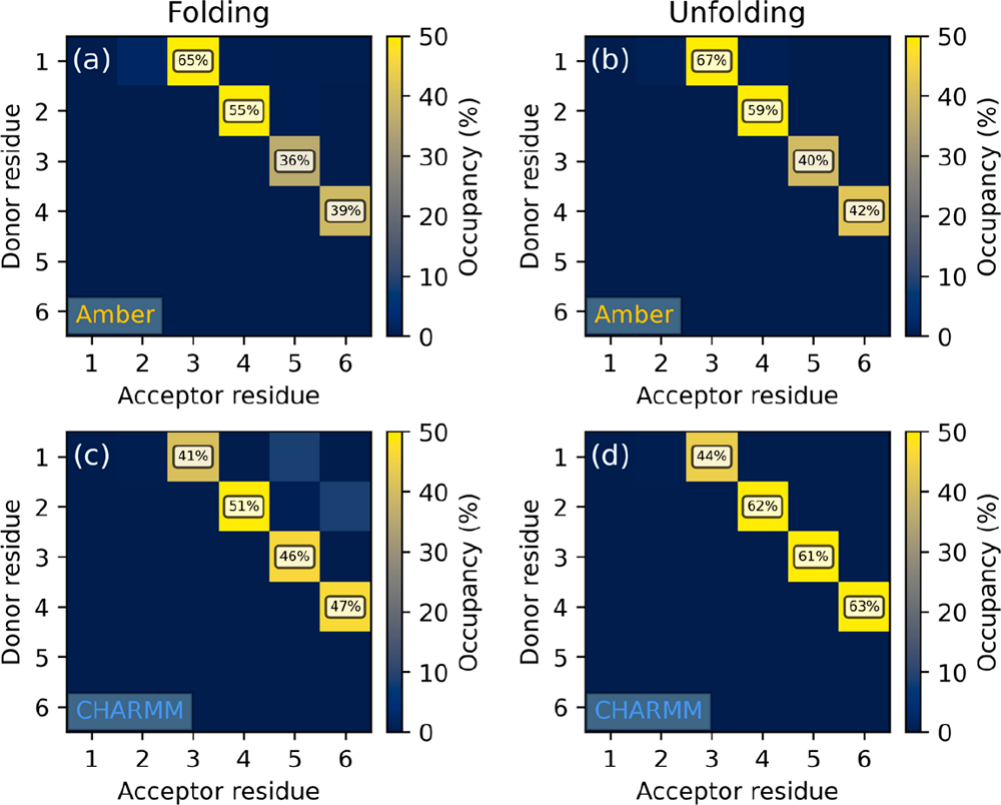
Hydrogen bond occupancy maps of peptide II simulated in water under the (a, b) Amber and (c, d) CHARMM FFs, started from (a, c) extended and (b, d) 3_14_ helix conformations. Occupancies below 10% are not labeled.

The occupancy analysis, based on all independent reruns, shows that both FFs sample the 3_14_ helix conformation with high statistical weight, CHARMM being slightly better in this respect (Figure 6). Partial unfolding is also permitted by both parameter sets, even with some of the backbone torsions being fixed by the cyclic β-amino acid residues at angles required for the 3_14_ helix. Interestingly, in simulation II/a (Figure 6c), an additional secondary structure characterized by *i* → *i* + 4 hydrogen bonding is also found, albeit with a very low statistical weight. This corresponds to the 18-membered pseudorings of the 4_18_ helix. An *ab initio* study on β-peptide helices by Möhle et al. reported that though this conformation is theoretically possible, it requires longer sequences and probably also some support from the solvent to stay stable, as a tetrapeptide prepared in the idealized 4_18_ geometry instantly refolded into the 3_14_ helix.^102^

**Figure 7:**
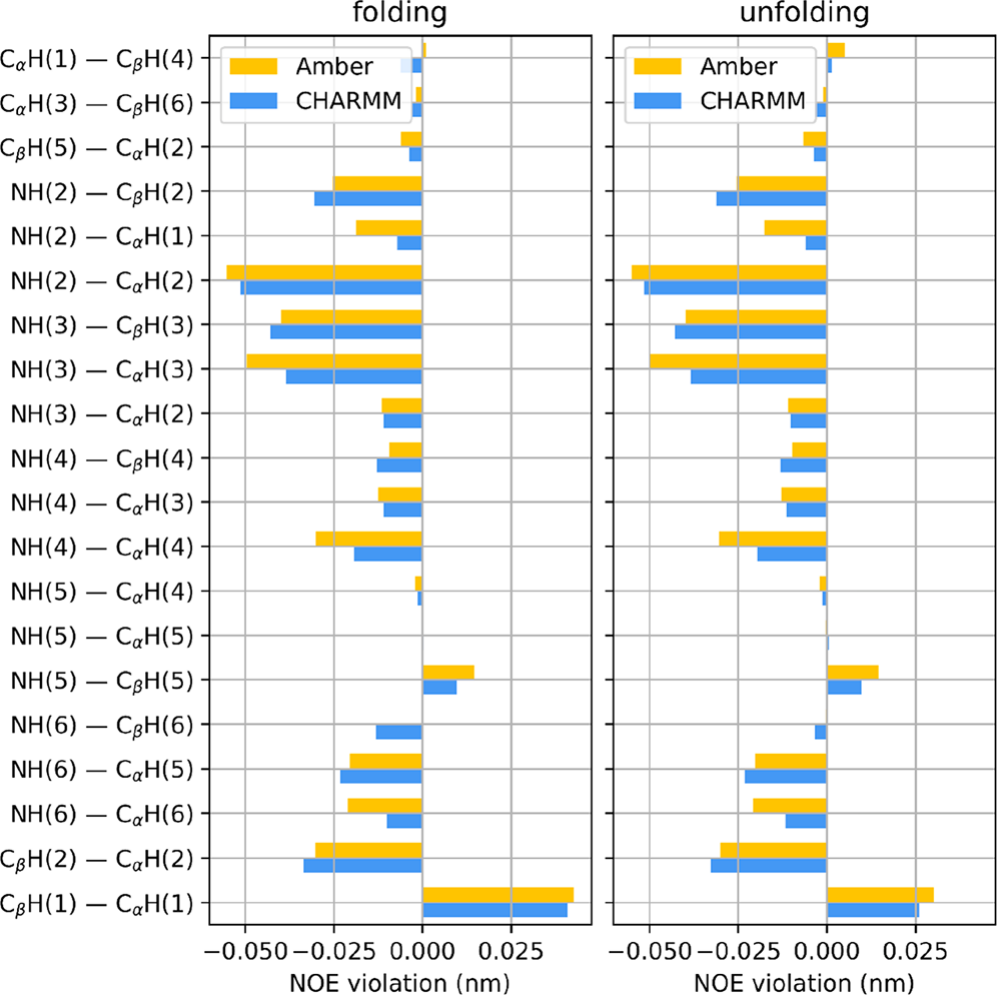
Effective violations of experimental hydrogen–hydrogen NOE distances for peptide II, started from (left) extended and (right) 3_14_ helical conformations. Averaging was done only on the last 300 ns of the trajectories.

With the help of NOE distance violations, the validity of the two force fields can be assessed. The results shown in Figure 7 indicate that both Amber and CHARMM give experimentally sane conformations (the largest upper bound violation being around 0.04 nm), with the latter FF yielding slightly closer matches.

Peptide III, in contrast to the previously discussed one, has no cyclic β-amino acid components and has been reported to have a random conformation in solution.^50^ The evolution of helicity over time (shown in Figures S22 and S23) also reflects this, showing frequent unfolding/refolding and a significantly shorter lifetime for both force fields, even for those trajectories where the time average of the helicity score was the largest.

**Figure 8:**
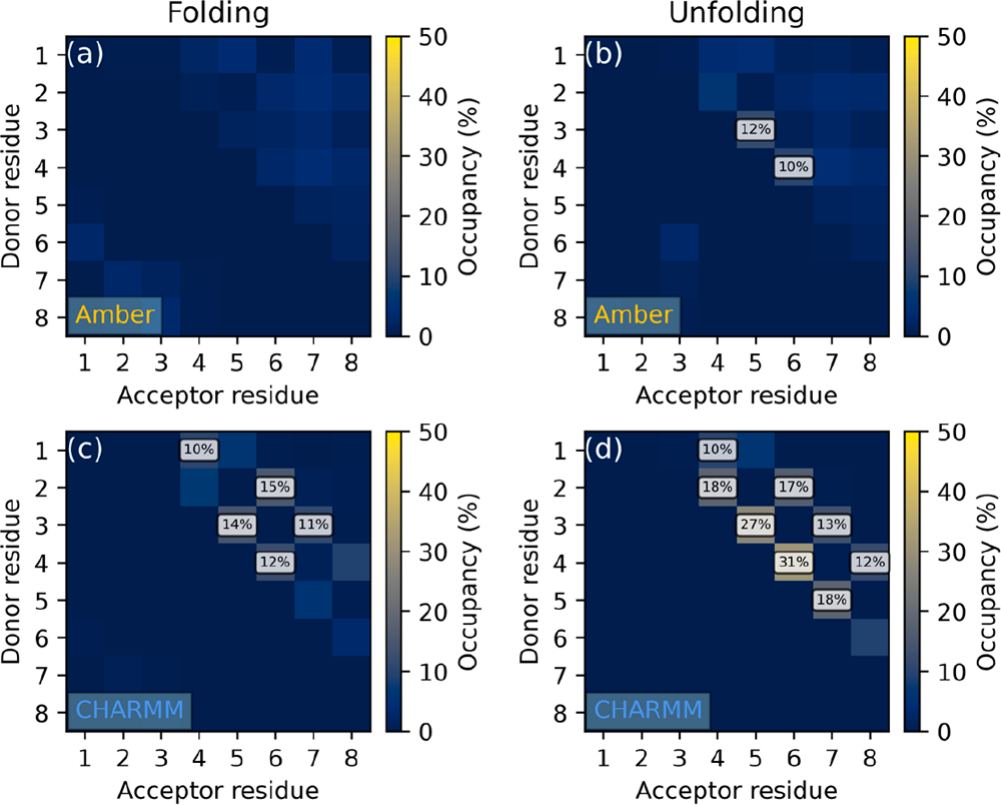
Hydrogen bond occupancy maps of peptide III simulated in water under the (a, b) Amber and (c, d) CHARMM FFs, started from (a, c) extended and (b, d) 3_14_ helix conformations. Occupancies below 10% are not labeled.

Intrachain hydrogen bond occupancy analysis results (Figure 8) show that regardless of the conformation the peptide was prepared in, the Amber FF keeps the peptide practically unfolded, while CHARMM recovers both 3_14_ and 4_18_ helices, albeit with a very small statistical weight. Note that here the performance in reproducing a helical structure is biased, as some backbone torsions are enforced by cyclic β-amino acids.

**Figure 9:**
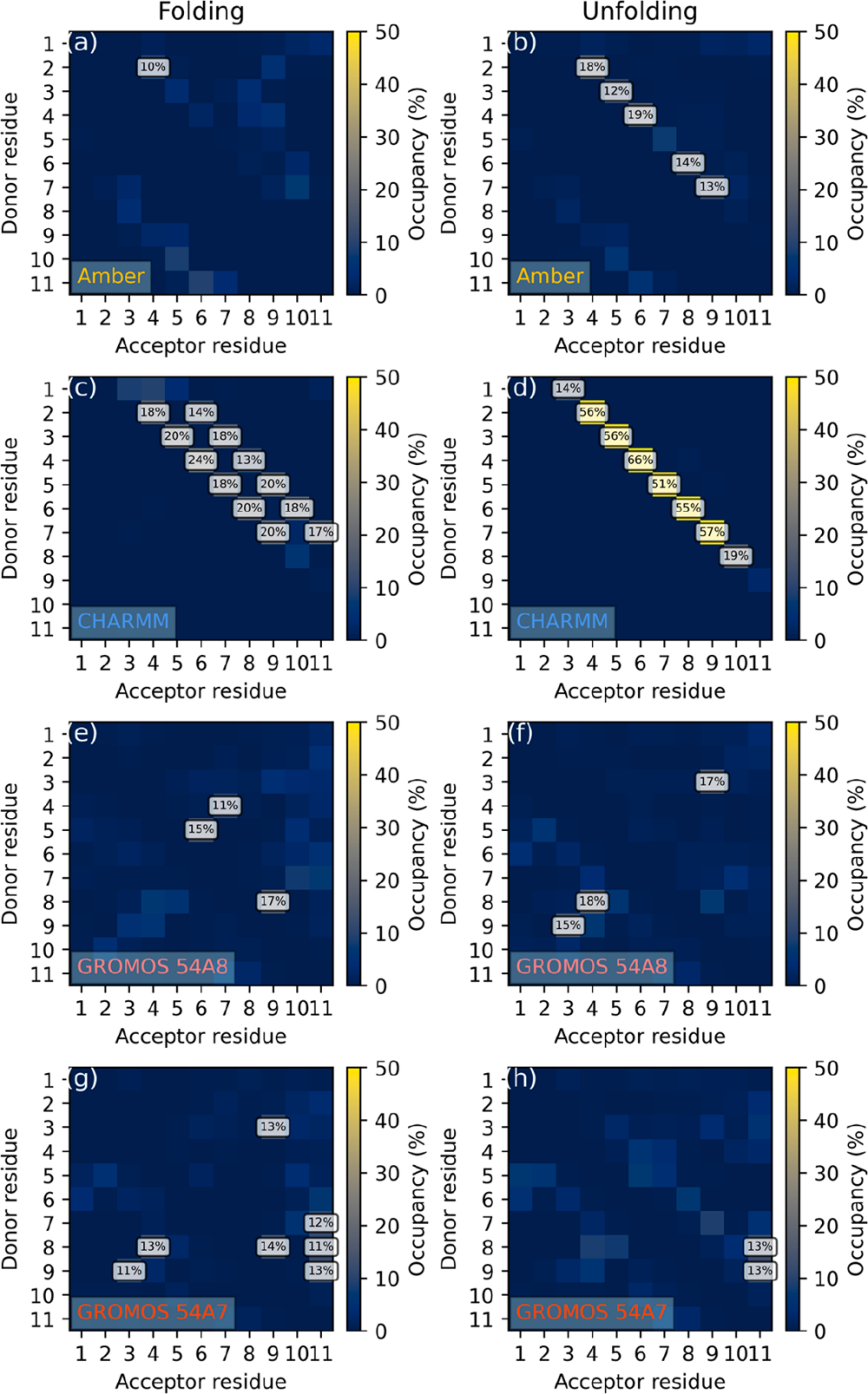
Hydrogen bond occupancy maps of peptide IV simulated in water under the (a, b) Amber, (c, d) CHARMM, (e, f) GROMOS 54A8, and (g, h) GROMOS 54A7 FFs, started from (a, c, e, g) extended and (b, d, f, h) 3_14_ helix conformations. Occupancies below 10% are not labeled.

We therefore performed simulations on peptide IV, which has been reported to form 14-helices in aqueous solution^60^ and consists entirely of acyclic β-amino acids. The intrachain hydrogen bond occupancy maps in Figure 9 show that the hydrogen bond pattern characteristic to the 3_14_ structure is clearly recovered by CHARMM regardless of the starting conformation. Additionally, a spontaneous folding into the 4_18_ helix is also seen, again confirming the surmise of Möhle et al. that in longer sequences this secondary structure might be more stable than in shorter ones.^102^ The Amber FF in contrast unfolds the peptide almost instantaneously (Figures S24 and S25) even when originally prepared in the 3_14_ helix. Finally, neither of the GROMOS force fields was able to recover any of the two helices described above, showing only disordered structure.

### 3.2 Conformational Analysis of the Hairpin Structure

The hairpin form adopted by peptide V is one of the first purposefully designed β-peptide secondary structures. The intentional choice of the absolute conformations of the composing amino acids ensures that the two ends of the peptide are nearly straight, connected by a preferred turn in the middle. This fold was found to be relatively stable in methanol experimentally,^14^ albeit not so rigidly as would be expected from a 3_14_ helix.

**Figure 10:**
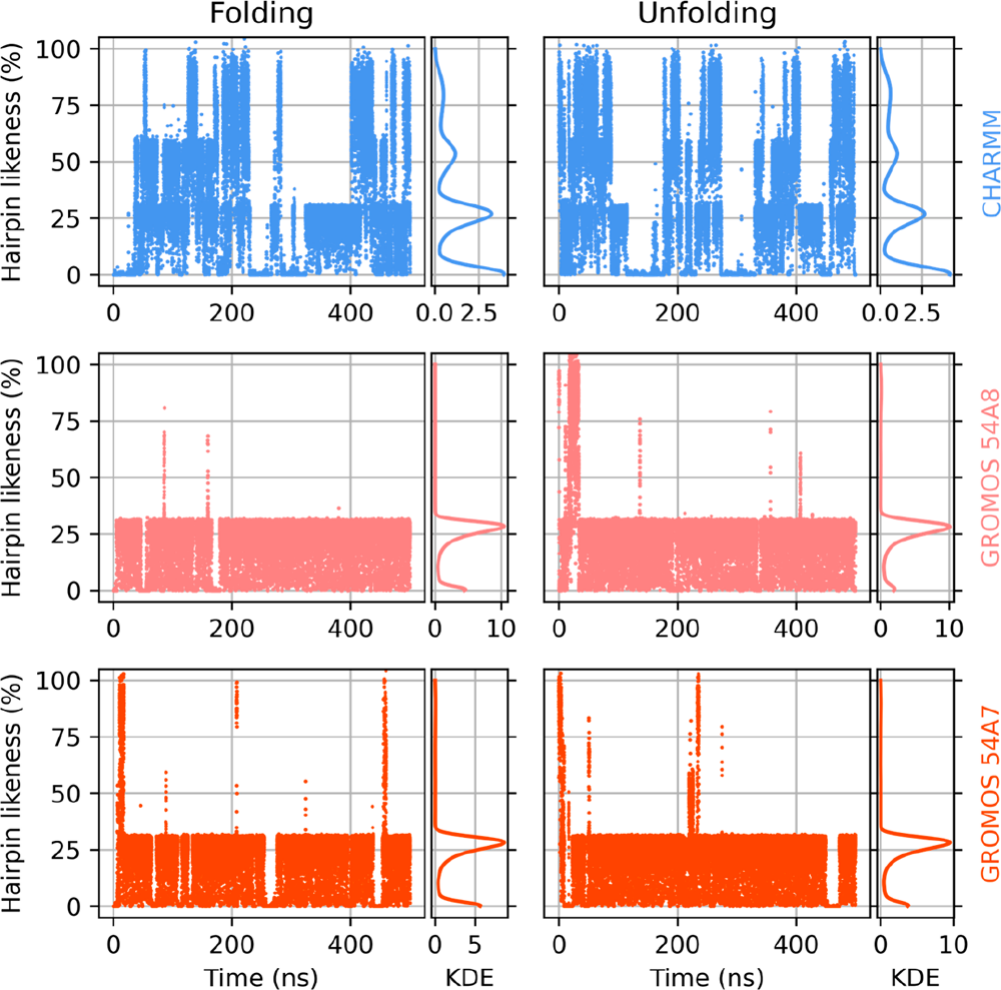
Evolution of the likeness score to the hairpin conformation of peptide V in methanol over time under CHARMM (top row), GROMOS 54A8 (center row), and GROMOS 54A7 (bottom row), when started from extended (left column) and the expected hairpin (right column) conformations. Gaussian kernel density estimates are shown to the right of the respective time series.

The conformational likeness score defined in eq 3 is plotted versus the simulation time in Figure 10. The GROMOS force fields mostly sample conformations with only one hydrogen bond from the three possible ones. On the other hand, CHARMM can reproduce a better hairpin conformation, with frequent “unzipping” and refolding of the structure.

**Figure 11:**
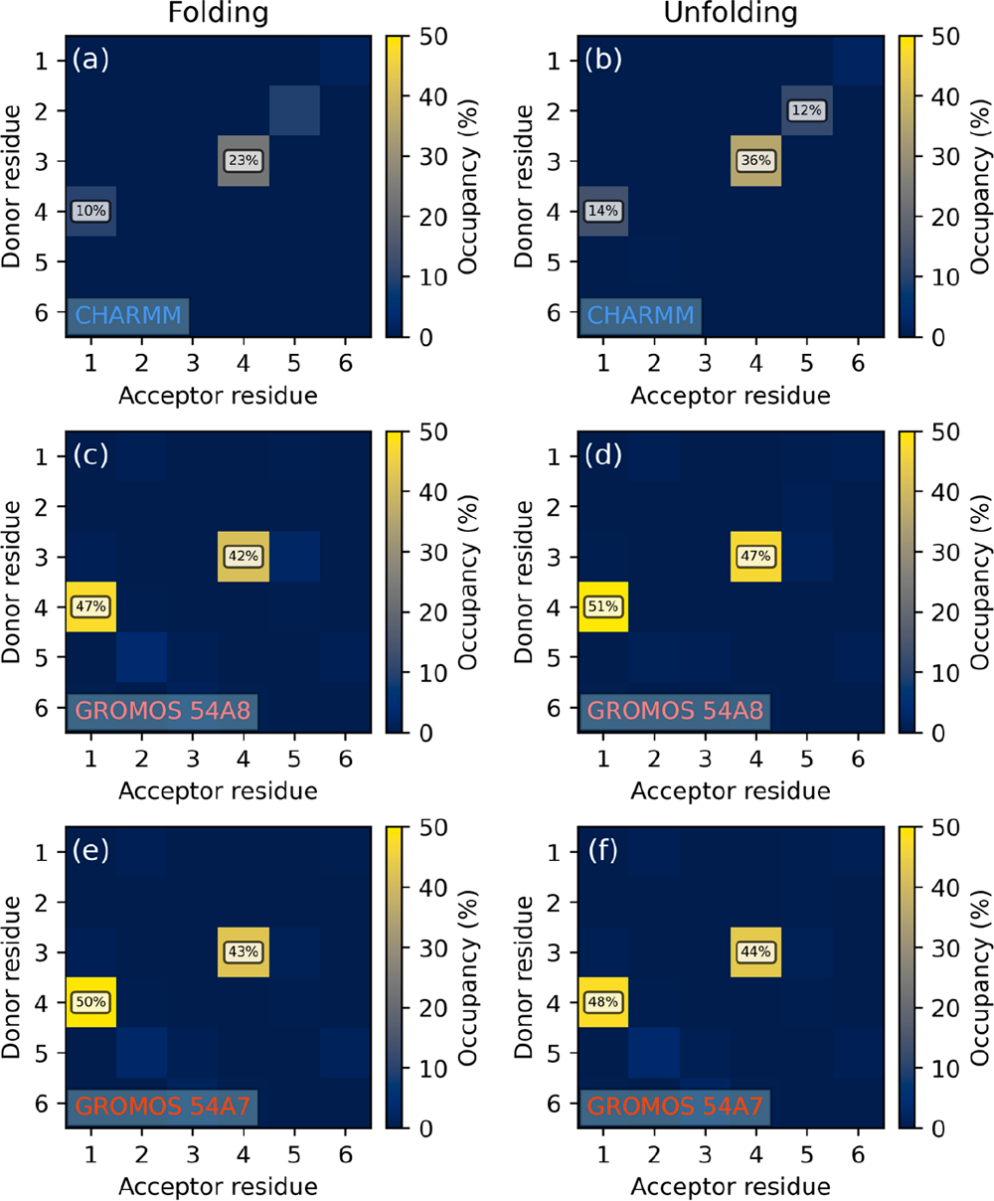
Hydrogen bond occupancy maps of peptide V simulated in methanol under the (a, b) CHARMM, (c, d) GROMOS 54A8, and (e, f) GROMOS 54A7 FFs, started from (a, c, e) extended and (b, d, f) the expected hairpin conformations. Occupancies below 10% are not labeled.

The results of occupancy analysis reflect the same properties (Figure 11). It is shown that the middle hydrogen bond (3 → 4) is well represented in all cases. This bond turned out to be more stable with the GROMOS force fields, with occupancy over 42% in all cases, whereas CHARMM reports only 23 and 36% occupancy. On the other hand, this force field recognizes the 2 → 5 bond in addition, although only with a very small occupancy, due to the inherent flexibility of the peptide sequence.

**Figure 12:**
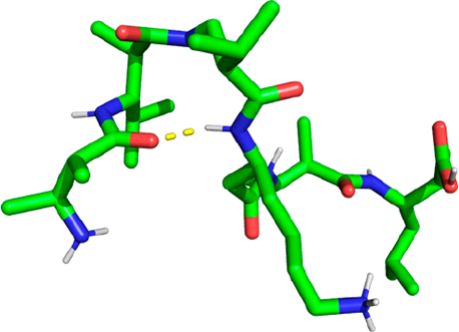
Representative geometry of peptide V with a hydrogen bond existing between residues 4 and 1.

Interestingly, another hydrogen bond involving the amide hydrogen from residue 4 and the amide oxygen from residue 1 also frequently occurs with both FFs, although to a much smaller extent with the former. This corresponds to a pseudoring of 12 atoms, characteristic to the 2.5_12_ helix, the second most stable helical conformation after 3_14_.^102^ The GROMOS force field samples this conformation (∼50%; the other two *i* → *i* – 3 hydrogen bonds are also seen) with a larger statistical weight than that for which the peptide was designed (∼40% for the 3 → 5 bond; the other two are nearly nonexistent). As with the hairpin structure, the rest of the molecule is still flexible, twisting and turning in a random fashion (a representative geometry is shown in Figure 12). The statistical weight of the two conformations is the other way around with the CHARMM FF (20–30% vs 10% hydrogen bond occupancy for the strongest hydrogen bond of the hairpin and the 2.5_12_ helix, respectively).

An analysis of NOE proton–proton distances on this peptide revealed a very good conformity to the experimentally determined distance bounds, studied with the CHARMM FF, as all but one of the upper NOE distance bound violations are negative in both directions of the simulation (folding/unfolding), with the single positive violation being on the order of 0.025 nm (Figure S38).

### 3.3 Oligomerization of Strand-Forming β-Peptides

Numerous β-peptides are prone to form associates, long fibrils, or helical oligomeric bundles in solution, driven by secondary interactions.^1,24,64^ Initial observations of these phenomena were made on fibrils and membranes formed by β-peptides composed of cyclic β-amino acid residues.^9,58^

One of our chosen model sequences, peptide VI, is reported to form aggregates in methanol and water. As this is a pentapeptide of five heterochiral 2-aminocyclopentanecarboxylic acid (ACPC) monomers, intrachain hydrogen bonding and thus helix formation are precluded, as the molecule prefers an extended conformation instead. This leaves the peptide bonds free to form interchain hydrogen bonds, contributing to the formation and stabilization of larger, strandlike aggregates.^50,58,59^

Single-chain simulations in DMSO, water, and methanol (VI/a–c in Table 1, respectively) confirm the absence of intrachain hydrogen bonds (Figures S11, S12, S14, and S15). Most of the NOE upper bound violations are negative for both Amber and CHARMM, in the case of all three solvents: methanol, DMSO, and water (Figure S39). Positive violations are on the order of ≤0.05 nm, the Amber force field performing somewhat better, yielding fewer and lower positive violation values.

Associate forming was explored by simulations VI/d and VI/e, where eight peptide chains were placed in a box of solvent, situated at the corners of a cube, in random orientation. After the simulations, the oligomers were detected at each time step using the modified Hoshen–Kopelman algorithm, the criterion of two chains belonging to the same associate being the existence of at least one hydrogen bond between them. Next, the lifetime of oligomers, i.e., the time in which the hydrogen bond network existed, was enumerated. Relevant statistics from dimer, trimer, and higher oligomer lifetimes are shown in Table 2.

**Table 2:** Lifetimes of Peptide IV Associates

system	associate	force field	median lifetime (ns)	longest lifetime (ns)	trajectory percentage	associates in 1000 ns
VI/d	dimer	Amber	0.05	2.70	10.3%	14587
		CHARMM	0.05	12.00	30.6%	17800
	trimer	Amber	0.02	0.13	0.1%	393
		CHARMM	0.04	0.56	0.7%	1610
VI/e	dimer	Amber	0.03	3.87	36.9%	82800
		CHARMM	0.04	5.81	18.4%	22913
	trimer	Amber	0.01	0.30	2.2%	10720
		CHARMM	0.02	0.50	0.4%	1240
	tetramer	Amber	0.01	0.08	0.1%	913
		CHARMM	0.01	0.03	<0.1%	67
	pentamer	Amber	0.01	0.01	<0.1%	40
		CHARMM	–	–	–	–

In methanol (system VI/d), significantly more associates are found with CHARMM: in around 30% of the time, at least one dimer exists, with the most stable dimer being associated for 12 ns. In contrast to this, the associates are shorter-living and scarcer under the Amber force field. The situation is reversed in water: dimers cover more than 36.9% of the trajectory with the Amber force field and only 18.4% with CHARMM. However, the associates are more stable under CHARMM, with both the median and longest lifetime being larger than in Amber.

**Figure 13:**
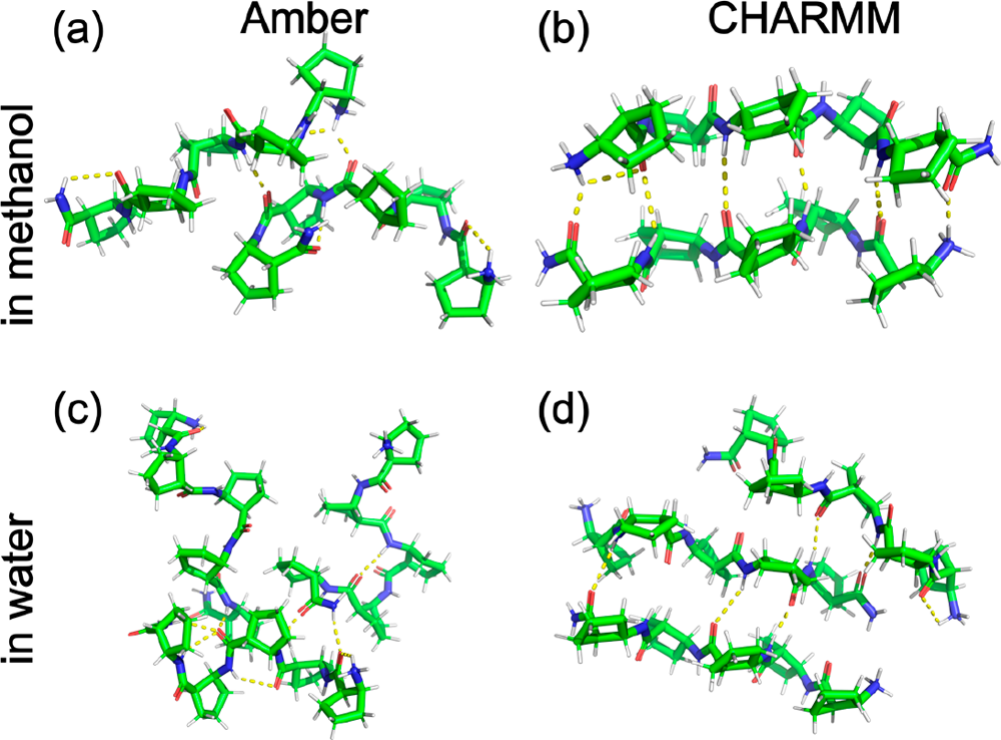
Representative associates of peptide VI in (a, b) methanol and (c, d) water, simulated using the (a, c) Amber and (b, d) CHARMM force fields.

The higher stability of the associates is readily understood by looking at representative geometries of these (Figure 13). In both methanol and water, the CHARMM FF produces more ordered associates, which are held together by more than one interchain hydrogen bond. Presumably, such associates act as structural building blocks of larger strand- and sheetlike aggregates observed in the literature.^59^ In this sense, CHARMM is more adept at recovering the structural driving force of observed aggregation behavior.

### 3.4 Stability of Zwit-EYYK Octamers

In contrast to peptide VI, the amides of peptide VII are occupied for the task of stabilizing the 3_14_ helices, and therefore, the associate is held together by salt bridges between positively and negatively charged side chains as well as by hydrophilic–hydrophobic effects, with the β-homoleucines forming a hydrophobic core.^1^

This peculiar method of stabilization through side chains makes the backbone regions more mobile in simulation, allowing the RMSD of these from the published crystal structure (CCDC 804687 in The Cambridge Crystallographic Data Center) to be used as an indirect indicator for the goodness of side-chain parametrization.

**Figure 14:**
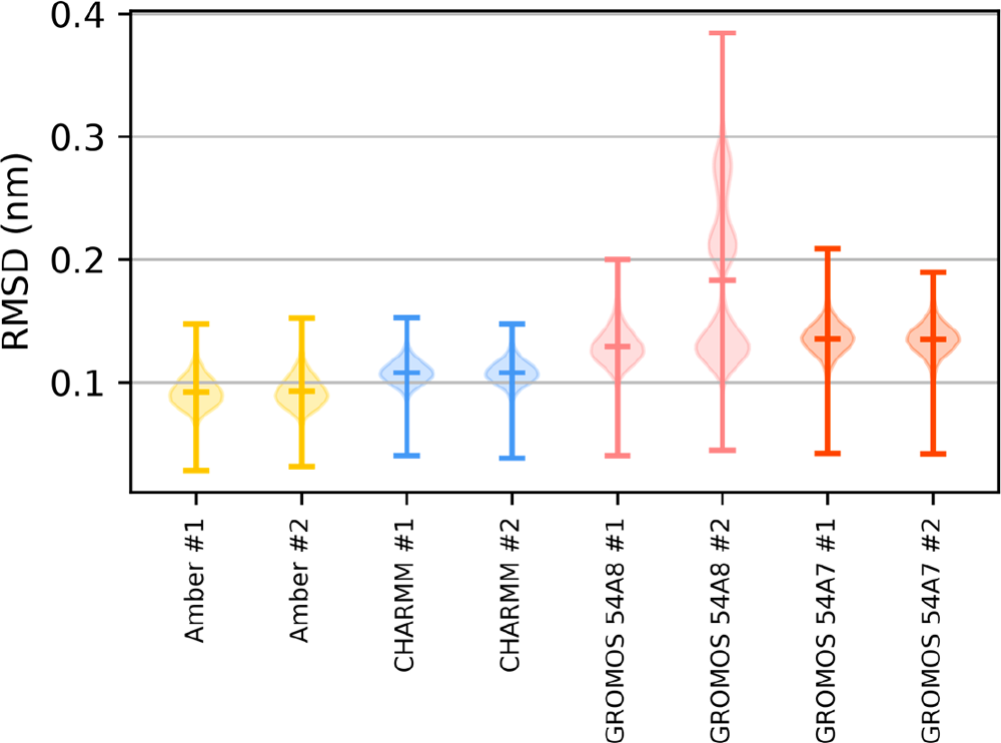
Violin plots of the root-mean-square deviation from the reference structure of peptide VII.

The required parameters and topologies are present in all three force fields, with the exception of the ornithine side chain, which is straightforward to derive from lysine by analogy. Two independent runs were performed with each of the four force field variants. Figure 14 shows violin plots, i.e., Gaussian kernel density estimates, as well as the median and the extrema of the RMSD of non-hydrogen atoms in the peptide backbone (the plots of RMSD over time are available in Figures S28–S35). Table 3 summarizes the mean and variance of the RMSD obtained with each FF on the aggregated trajectories of all independent reruns of the same system.

**Table 3:** Root-Mean-Square Deviation of the β-Peptide Backbone from the Reference Structure

force field	mean RMSD (nm)	std RMSD (nm)
Amber	0.093	0.012
CHARMM	0.108	0.009
GROMOS 54A8	0.156	0.052
GROMOS 54A7	0.136	0.012

The similarity of the two independent repetitions shows that the differences between force fields are systematic. Amber gives the lowest RMSD, the sampled ensemble being closer to the crystal structure. CHARMM gives slightly worse results in this case. The GROMOS force fields yield the largest deviations, the second repetition with GROMOS 54A8 additionally resulting in partial falling apart of the structure.

### 3.5 Performance Considerations

Simulations were performed on single nodes of a heterogeneous cluster, all equipped with NVidia 1080Ti graphical coprocessors, using version 2019.5 of the GROMACS software. A general observation was that the running time strongly depended on the cutoff distances of Coulomb and van der Waals forces. Since these values are dictated by the force field parametrization, the computing performance turns out to be an important factor in choosing a FF. On an Intel Core i7-7700 CPU, simulations with Amber (0.8 nm cutoff) were significantly faster (ca. 370 ns/day for simulation III/a in Table 1.) than with CHARMM (1.2 nm, ca. 215 ns/day). The speedup that would have been obtained from the reduced number of atoms in the united atom force field GROMOS was defeated by the large cutoff distance of 1.4 nm (ca. 114 ns/day). Additionally, as some molecules have cyclic constraints in this parametrization, the well-parallelizable LINCS constraint algorithm could not be used, only the much slower, but more general SHAKE algorithm, further contributing to the reduction in the throughput.

### 3.6 Convergence of the Trajectories

An important but frequently neglected quality measure of an MD simulation is the question of convergence, i.e., whether a stationary state has been achieved after an initial transient period. Several ways exist for assessing convergence,^103−107^ the simplest approach of which is a qualitative analysis of the evolution of physical quantities relevant to the studied process or structure, such as conformity scores with respect to a target conformation, as we have employed for the 3_14_ helix and the hairpin fold. A more general measure, not tied to only one dominant conformational state, is the pairwise root-mean-square difference, on which conformational clustering can be based. Here convergence is said to have been attained if all conformational states have been explored (which also raises the question of ergodicity) and the sampling probability of the states becomes independent of time. For highly flexible molecules, such as most of our peptides, the required simulation time to satisfy the above criteria is prohibitively large due to the low transition rates between the states. Additionally, the ability to obtain the dominant conformation of a peptide in a reasonably short time is an important performance factor. Therefore, we used the simpler approach by Smith and co-workers,^103^ namely, defining convergence by the plateauing of the cumulative count of conformational clusters during the simulation. When the number of representative conformations required for classifying the trajectory stops increasing, the simulation is thought to have visited the most important secondary structures. We used the clustering method by Daura et al.,^108^ using C_α_ atoms of the central residues to calculate the RMSD, with a cutoff distance of 0.1 nm (the saturation curves are shown in the Figures S40–S46). Performing several independent reruns of the same system also worked toward enhancing the sampling quality. Most runs did show a plateau, especially for β-peptides containing cyclic residues. Some simulations were found not to be convergent or to start off exploring other conformational clusters after a relatively long plateau. However, due to the requirement of achieving the experimental fold in a reasonable short time and the aforementioned low transition rates between folds, longer simulations are not expected to come with significant benefits. The more so, as it is readily seen even on the selected time scale whether a particular force field is able to fold the peptide or not.

## 4 Conclusions

In the present work, the performances of three force field families in which specific modifications were made to improve reproduction of β-peptide structures were compared with respect to predicting and recovering β-peptide folding and association.

In terms of applicability, the CHARMM extension previously developed by us^54^ turned out the most versatile, supporting a wide range of singly and doubly substituted β-amino acids with all proteinogenic side chains, as well as two cyclic β-residues. This is enhanced by the underlying FF, implementing a wide range of chain terminating groups and a large subset of the chemical molecule space. This was a shortcoming of the other two force fields, where the absence of certain termini or residue topologies made it impossible to handle some of the seven β-peptides without modification or reparametrization: Amber could only be applied for peptides II, III, VI, and VII, while the GROMOS FFs were only applicable for peptides I, IV, V, and VII. These two force fields could not therefore be evaluated for all systems.

The parametrization method of a particular force field, i.e., the delicate balance of backbone torsions and the electrostatic and van der Waals potentials, turned out to be of utmost importance. In terms of predicting β-peptide secondary structure, the Amber and CHARMM force fields were able to spontaneously fold peptide II into its experimentally validated 3_14_ helical conformation, with the former FF doing so slightly faster than the latter. It also correctly reports a disordered structure for peptide III. However, this behavior turned out to depend on the presence or absence of forced backbone torsions due to cyclic β-amino acids (*trans*-ACHC). The Amber FF, where only the partial charges of atoms are fitted but the dihedral parameters are taken by analogy, cannot fold peptide IV into its expected helical structure. Moreover, it also failed to keep the experimental helical conformation stable when that was used as the starting conformation. In contrast, the CHARMM extension, derived through a rigorous parametrization of the dihedral potential energy surface, is found to obtain reasonable folding statistics: spontaneously folding all peptides which were reported to have a helical structure (I, II, IV) when starting from an extended conformation. Conversely, the experimentally known structure was preserved when the peptide was prepared in it at the start of the simulation. In addition, alternative folds were also sampled beside the dominant folds, such as the negative 18-helix (peptides II, III, and IV) and the positive 12-helix (peptide V). The dynamic equilibrium with partial unfolding and refolding hints that this FF can model a balanced system, without an artificial preference of any conformation.

Somewhat contrary to our expectations, although β-peptides are supported out of the box, we were not able to predict the correct folding behavior of any peptide with the GROMOS FF, unfortunately not even those which were initially used for validation during development of the force field.^63,69^ We strongly suspect that here the serious algorithmic differences between the GROMOS FF and the recent versions of the GROMACS MD engine are to blame.^97,98,100^ This is even more plausible, as the differences mentioned above manifest in the parametrization of Coulombic and van der Waals interactions, which are the primary actors controlling hydrogen bonding behavior in MD simulations.

Studies of spontaneous self-assembly revealed that simulations with the CHARMM force field always find more ordered associates that remain stable for longer time periods than with the Amber parameters. This is probably once again caused by the correctly determined backbone potentials, allowing the peptide to adopt the required conformations, which results in a better stacking of the monomers, stabilized by multiple hydrogen bonds between each pair of molecules within the oligomers.

For five of the investigated peptides, NMR- and X-ray-based structural information was available. Due to the obvious unfolding of the peptide conformation under them, the two investigated variants of the GROMOS force field could not be tested in terms of experimental data. When comparing the relative performance of the other two force fields on the tested systems, CHARMM yielded values that are close to the experimentally observed ones in all five cases. Wherever the peptide sequence permitted treatment by the Amber force field, the conformity to NMR distance restraints was on par with what observed with CHARMM, in some cases yielding slightly better results, such as in the case of the peptide VII octameric associate, where the Amber force field reported the lowest root-mean-square deviation from the experimental structure determined by X-ray diffraction, with CHARMM performing second-best. This aspect will be increasingly important in the future of the currently available force fields as non-natural peptides have a high potential to enter their second golden age with assembly formation playing a key role from soft matters in nanotechnology to supramolecular coassemblies with biomedical relevance.

## Data Availability

Three-dimensional models of the peptides were created with PyMOL,^79^ employing the pmlbeta plug-in.^80^ The coordinates of the peptide VII octamer were obtained the Cambridge Crystallographic Database, deposition number 804687. Molecular dynamics simulations were performed with version 2019.5 of GROMACS.^77^ Calculations were orchestrated using the self-developed gmxbatch library (https://gitlab.com/awacha/gmxbatch). Trajectories were evaluated and figures were drawn using Jupyter notebooks,^109^ employing the NumPy,^110^ SciPy,^111^ Matplotlib,^112^ and MDAnalysis^92,93^ libraries. Chemical structures were drawn with jchempaint (https://jchempaint.github.io) and Inkscape (https://inkscape.org). All scripts, molecular dynamics input files, initial coordinates, and relevant simulation results are available at 10.5281/zenodo.7615256.

## Supporting Information Available

Details on the rederivation of the Amber force field parameters for β-peptides; molecular dynamics parameters used; intrachain hydrogen bond occupancy maps; time evolution of the helicity score and the hairpin conformation likeness score for all simulations performed; RMSD from the reference crystal structure of the octameric bundle of peptide VII; NOE distance limit violations for peptides I, II, V, and VI; cumulative cluster counts (PDF)
SMILES strings (TXT)

## Abbreviations

MD: molecular dynamics
MM: molecular mechanics
ACPC: 2-aminocyclopentanecarboxylic acid
ACHC: 2-aminocyclohexanecarboxylic acid
DMSO: dimethyl sulfoxide
FF: force field
