# Supplementary material for: Comparative Study of Molecular Mechanics Force Fields for β-Peptidic Foldamers: Folding and Self-Association
Source: J Chem Inf Model. 2023 Jun 6;63(12):3799–813. doi: 10.1021/acs.jcim.3c00175 (PMC10302482; doi:10.1021/acs.jcim.3c00175)
Supplement: Supplementary file 1 — ci3c00175_si_001.pdf [file ci3c00175_si_001.pdf]

Supplementary Information: Comparative Study of Molecular Mechanics of Force Fields for β-peptidic Foldamers: Folding and Self-Association

András Ferenc Wacha* wacha.andras@ttk.hu
Zoltán Varga
Tamás Beke-Somfai† beke-somfai.tamas@ttk.hu

Institute of Materials and Environmental Chemistry,
Research Centre for Natural Sciences,
Loránd Eötvös Research Network,
H-1117 Budapest, Magyar tudósok körútja 2.

1 Parametrization of β-amino acid residues for the Amber FF

β-amino acid residues were parametrized using *antechamber* [1], following the work of Németh et. al. [2], using the following steps:

1. Initial models were created using PyMOL [3, 4], with the `pmlbeta` plug-in developed by the authors of the present work [5]. For each residue, three molecules were created: one with Ace and NMe caps (mid-chain residue), one with NH_3^+ and NMe (N-terminal residue) and one with Ace and COO^- ending (C-terminal residue). Singly substituted β^3- and β^2-amino acids of all non-cyclic proteinogenic side-chains were created, as well as some disubstituted $\beta^{2,3}$-amino acids with a methyl group on the α carbon. Additionally, all stereoisomers of aminocyclopentanecarboxylic acid (ACPC) and aminocyclohexanecarboxylic acid (ACHC) were also created with all three capping variants.

2. For each variant of each amino-acid, *antechamber* was used to generate an input file for the Gaussian ab initio quantum chemistry program for geometrical optimization and generating electrostatic potential data for partial charge determination using the RESP method [6].

3. RESP charge fitting was done using the resp program supplied with AmberTools version 19. Charges on the Ace and NMe caps were constrained to their standard values found in the Amber force field.

4. Atom and bond type assignment was done by antechamber using the 'amber' scheme, compatible with FF99SB.

*corresponding author — calculations, methodology
†corresponding author — scientific topics, β-peptides

2 Molecular dynamics parameters

	Amber	CHARMM	GROMOS
rlist	0.8 nm	1.2 nm	1.4 nm
rcoulomb	0.8 nm	1.2 nm	1.4 nm
rvdw	0.8 nm	1.2 nm	1.4 nm
rvdw_switch	0 nm	1.0 nm	0 nm
coulombtype	PME	PME	PME
vdwtype	cutoff	cutoff	cutoff
vdw_modifier	none	force-switch	potential-shift-Verlet
DispCorr	no	no	EnerPres
constraints	H-bonds	H-bonds	all-bonds
Constraint algorithm	LINCS	LINCS	SHAKE

References

[1] Wang, J.; Wang, W.; Kollman, P. A.; Case, D. A. Automatic Atom type and Bond Type Perception in Molecular Mechanical Calculations. *J. Mol. Graph. Model.* **2006**, *25* (2), 247-260. https://doi.org/10.1016/j.jmgm.2005.12.005.

[2] Németh, L. J.; Hegedüs, Z.; Martinek, T. A. Predicting Order and Disorder for β-Peptide Foldamers in Water. *J. Chem. Inf. Model.* **2014**, *54* (10), 2776–2783. https://doi.org/10.1021/ci5003476.

[3] DeLano, W. The PyMOL Molecular Graphics System. http://www.pymol.org (accessed 2016-01-12).

[4] Schrödinger, LLC. The PyMOL Molecular Graphics System, Version 2.3.0; **2019**.

[5] Wacha, A.; Beke-Somfai, T. PmlBeta: A PyMOL Extension for Building β-Amino Acid Insertions and β-Peptide Sequences. *SoftwareX* **2021**, *13*, 100654. https://doi.org/10.1016/j.softx.2020.100654.

[6] Bayly, C. I.; Cieplak, P.; Cornell, W.; Kollman, P. A. A Well-Behaved Electrostatic Potential Based Method Using Charge Restraints for Deriving Atomic Charges: The RESP Model. *J. Phys. Chem.* **1993**, *97* (40), 10269–10280. https://doi.org/10.1021/j100142a004.

3 Intra-chain Hydrogen Bond Occupancy Maps

3.1 Folding of Peptide I in MeOH into 3_{14} helix

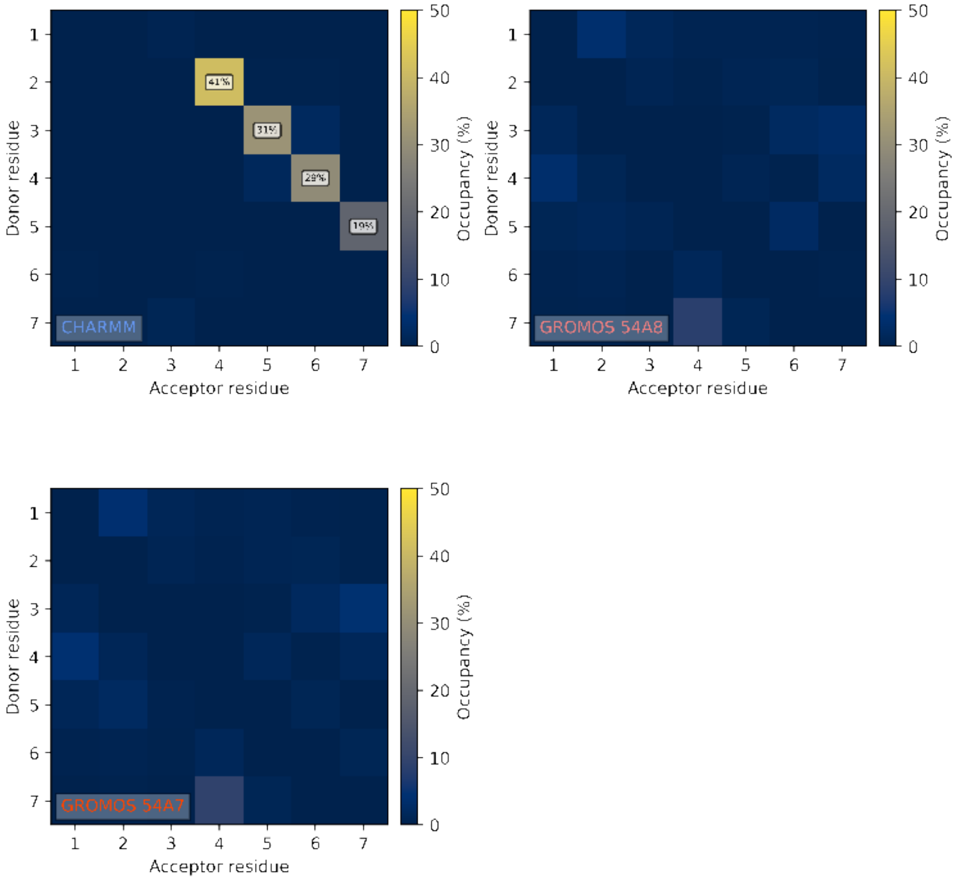

Figure S1: Intra-chain hydrogen bond occupancy map in the simulation about Folding of Peptide I in MeOH into 3_{14} helix. Only occupancies greater than 10% are labeled.

3.2 Unfolding of Peptide I in MeOH from 3_{14} helix

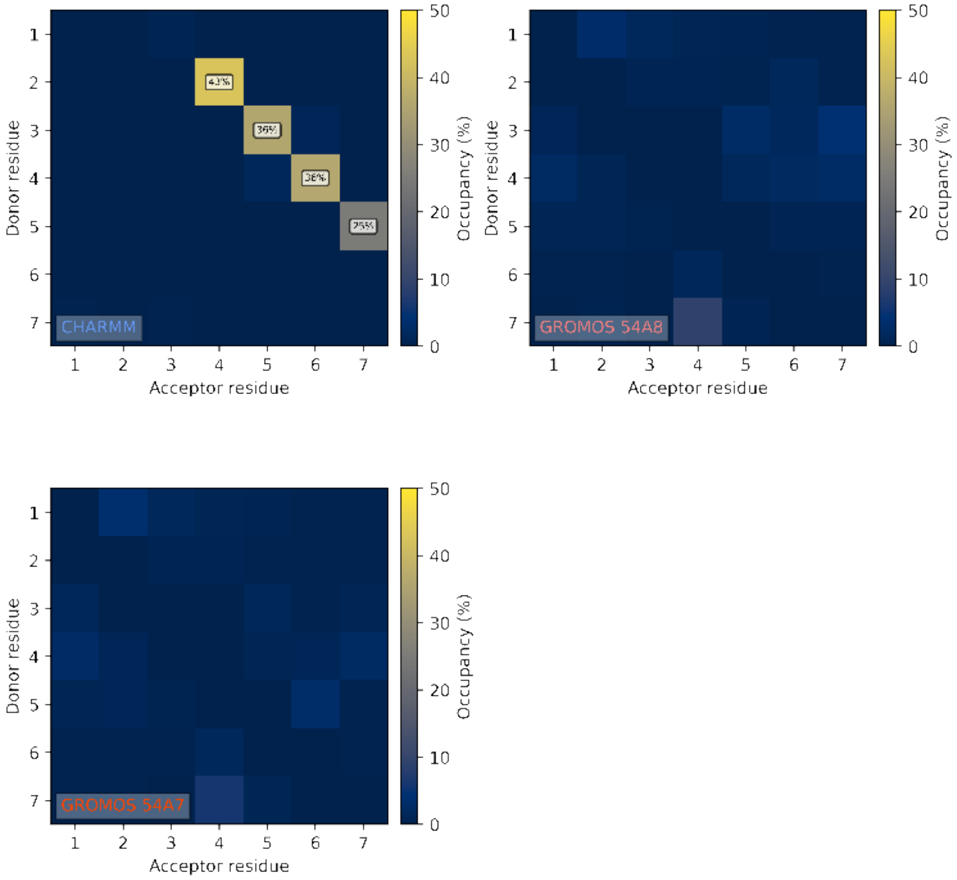

Figure S2: Intra-chain hydrogen bond occupancy map in the simulation about Unfolding of Peptide I in MeOH from 3_{14} helix. Only occupancies greater than 10% are labeled.

3.3 Unfolding of Peptide II in water from 3_{14} helix

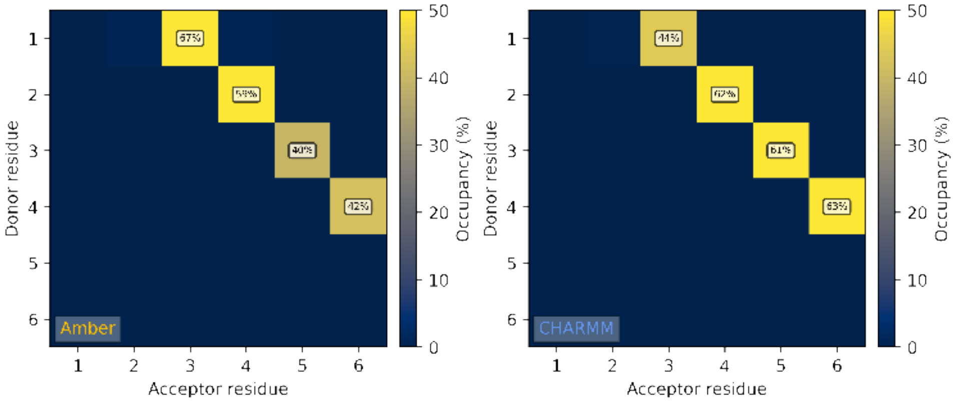

Figure S3: Intra-chain hydrogen bond occupancy map in the simulation about Unfolding of Peptide II in water from 3_{14} helix. Only occupancies greater than 10% are labeled.

3.4 Folding of Peptide II in water into 3_{14} helix

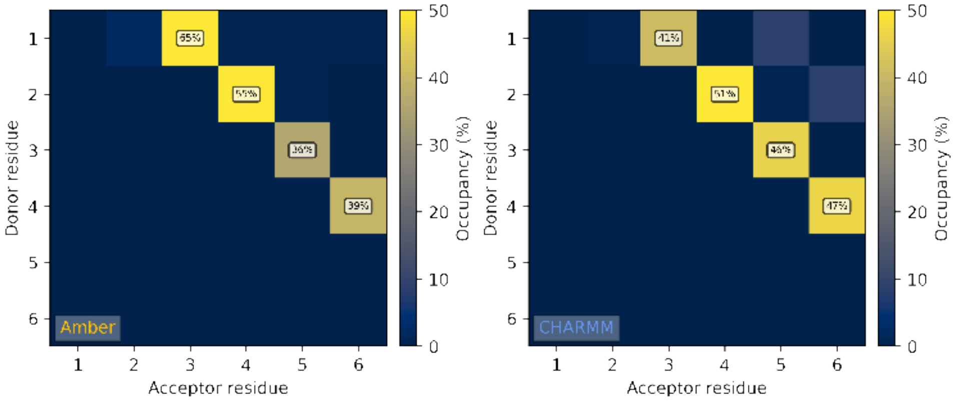

Figure S4: Intra-chain hydrogen bond occupancy map in the simulation about Folding of Peptide II in water into 3_{14} helix. Only occupancies greater than 10% are labeled.

3.5 Unfolding of Peptide III in water from 3_{14} helix

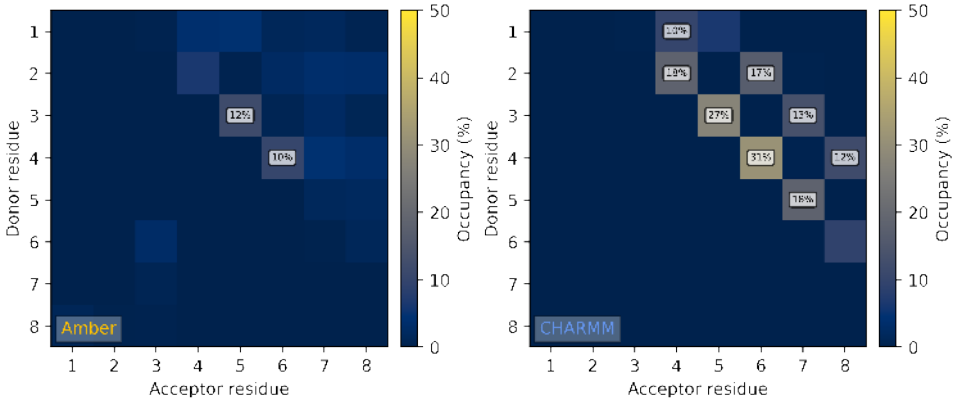

Figure S5: Intra-chain hydrogen bond occupancy map in the simulation about Unfolding of Peptide III in water from 3_{14} helix. Only occupancies greater than 10% are labeled.

3.6 Folding of Peptide III in water into 3_{14} helix

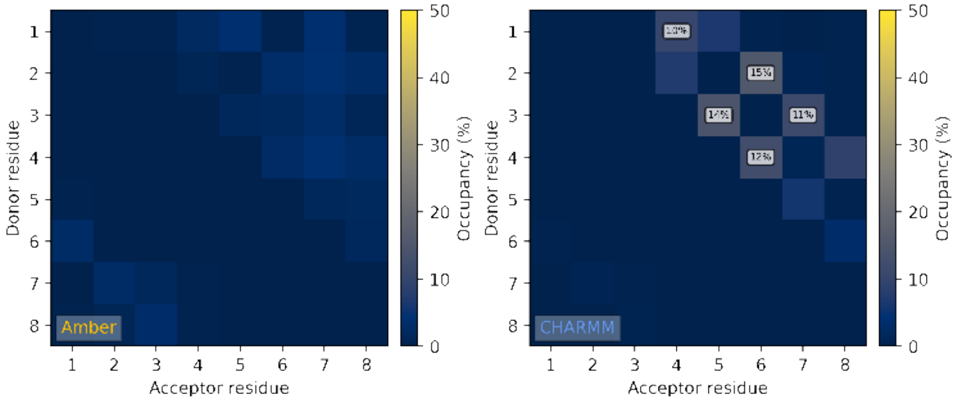

Figure S6: Intra-chain hydrogen bond occupancy map in the simulation about Folding of Peptide III in water into 3_{14} helix. Only occupancies greater than 10% are labeled.

3.7 Unfolding of Peptide IV in water from 3_{14} helix

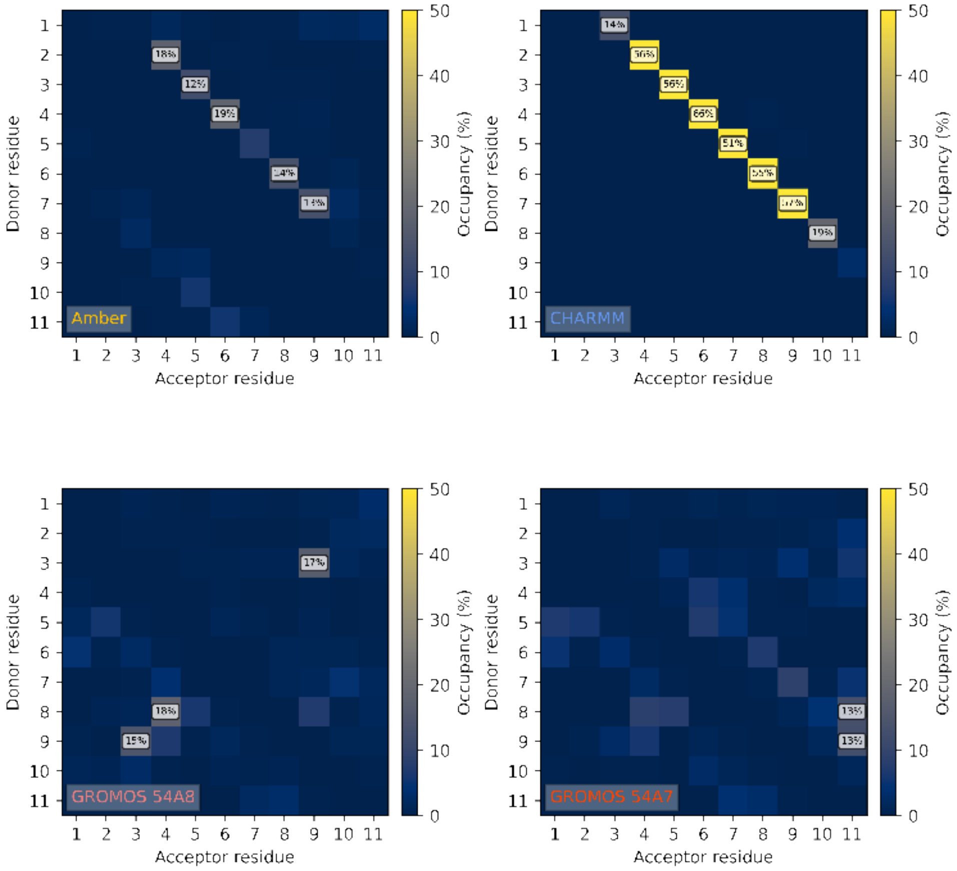

Figure S7: Intra-chain hydrogen bond occupancy map in the simulation about Unfolding of Peptide IV in water from 3_{14} helix. Only occupancies greater than 10% are labeled.

3.8 Folding of Peptide IV in water into 3_{14} helix

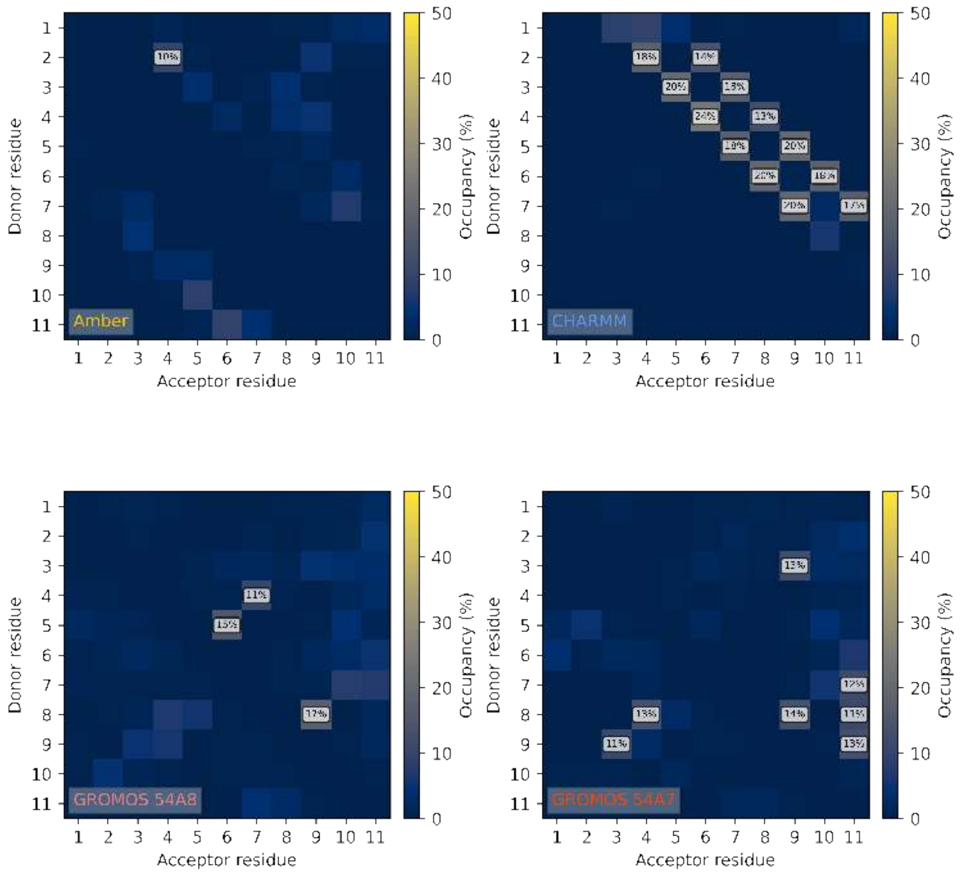

Figure S8: Intra-chain hydrogen bond occupancy map in the simulation about Folding of Peptide IV in water into 3_{14} helix. Only occupancies greater than 10% are labeled.

3.9 Unfolding of Peptide V in MeOH from hairpin conformation

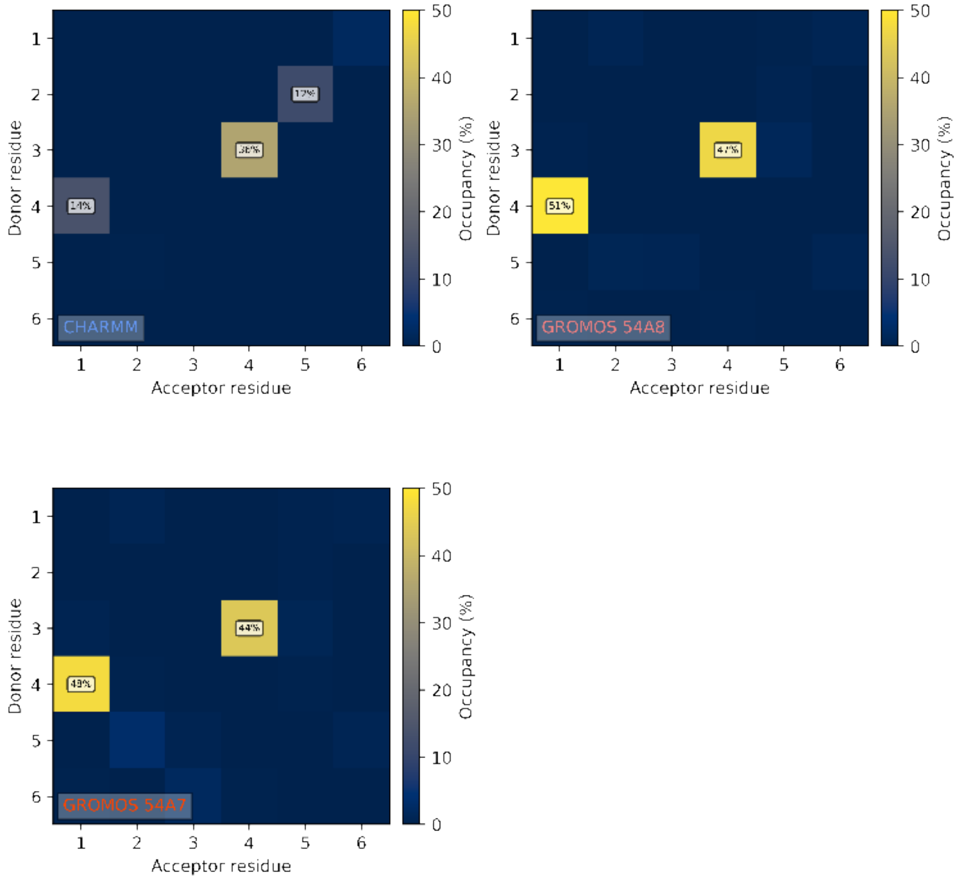

Figure S9: Intra-chain hydrogen bond occupancy map in the simulation about Unfolding of Peptide V in MeOH from hairpin conformation. Only occupancies greater than 10% are labeled.

3.10 Folding of Peptide V in MeOH into hairpin conformation

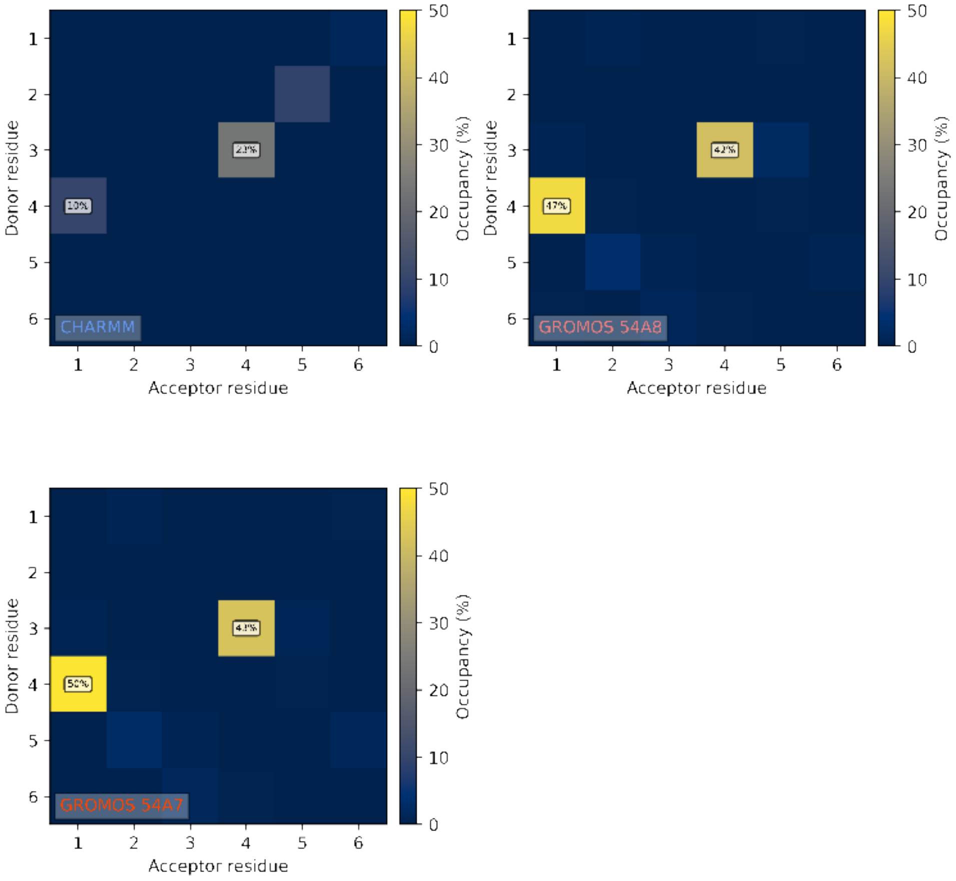

Figure S10: Intra-chain hydrogen bond occupancy map in the simulation about Folding of Peptide V in MeOH into hairpin conformation. Only occupancies greater than 10% are labeled.

3.11 Folding of Peptide VI in water

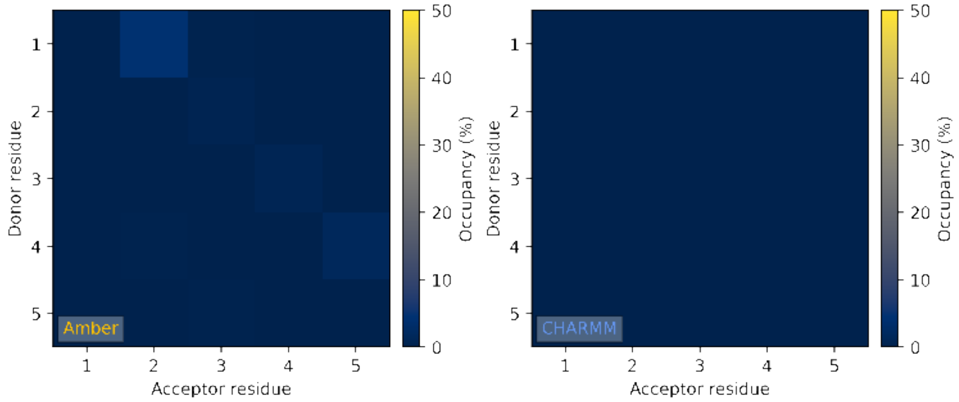

Figure S11: Intra-chain hydrogen bond occupancy map in the simulation about Folding of Peptide VI in water. Only occupancies greater than 10% are labeled.

3.12 Folding of Peptide VI in DMSO

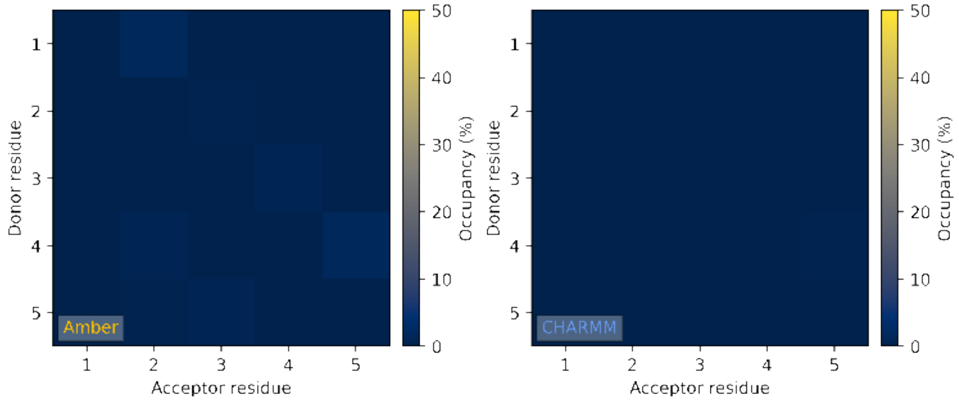

Figure S12: Intra-chain hydrogen bond occupancy map in the simulation about Folding of Peptide VI in DMSO. Only occupancies greater than 10% are labeled.

3.13 Folding of Peptide VI in MeOH

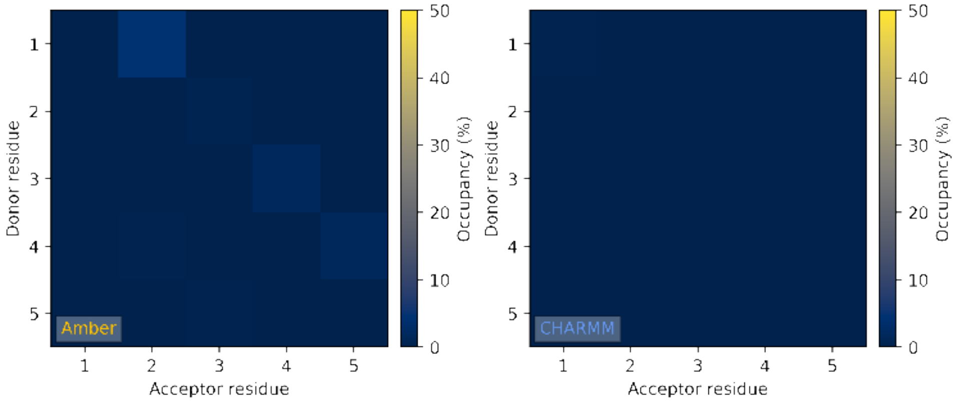

Figure S13: Intra-chain hydrogen bond occupancy map in the simulation about Folding of Peptide VI in MeOH. Only occupancies greater than 10% are labeled.

3.14 Aggregation of Peptide VI in water

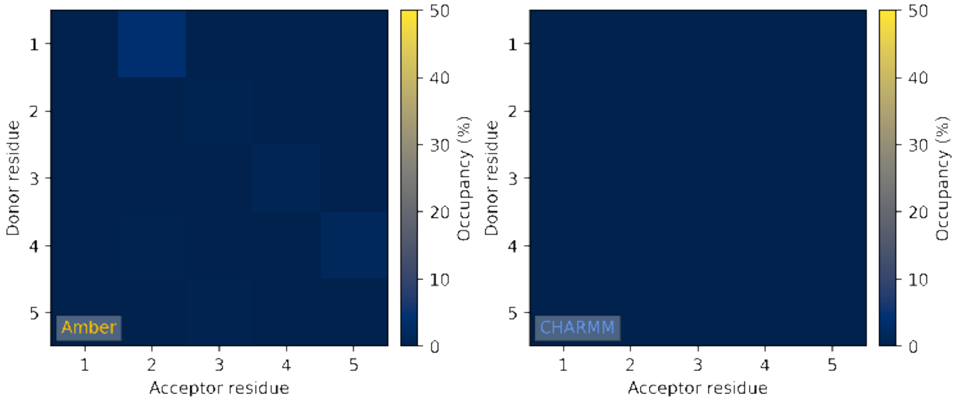

Figure S14: Intra-chain hydrogen bond occupancy map in the simulation about Aggregation of Peptide VI in water. Only occupancies greater than 10% are labeled.

3.15 Aggregation of Peptide VI in MeOH

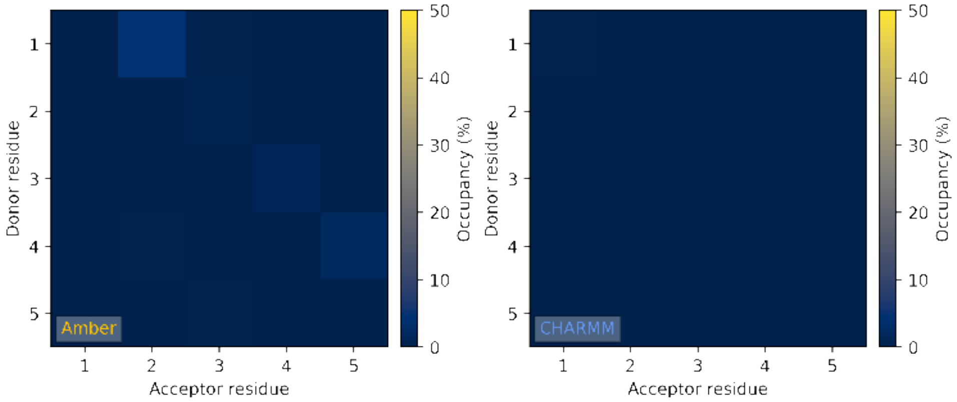

Figure S15: Intra-chain hydrogen bond occupancy map in the simulation about Aggregation of Peptide VI in MeOH. Only occupancies greater than 10% are labeled.

3.16 Unfolding of a single strand of Peptide VII in water from 3_{14} helix

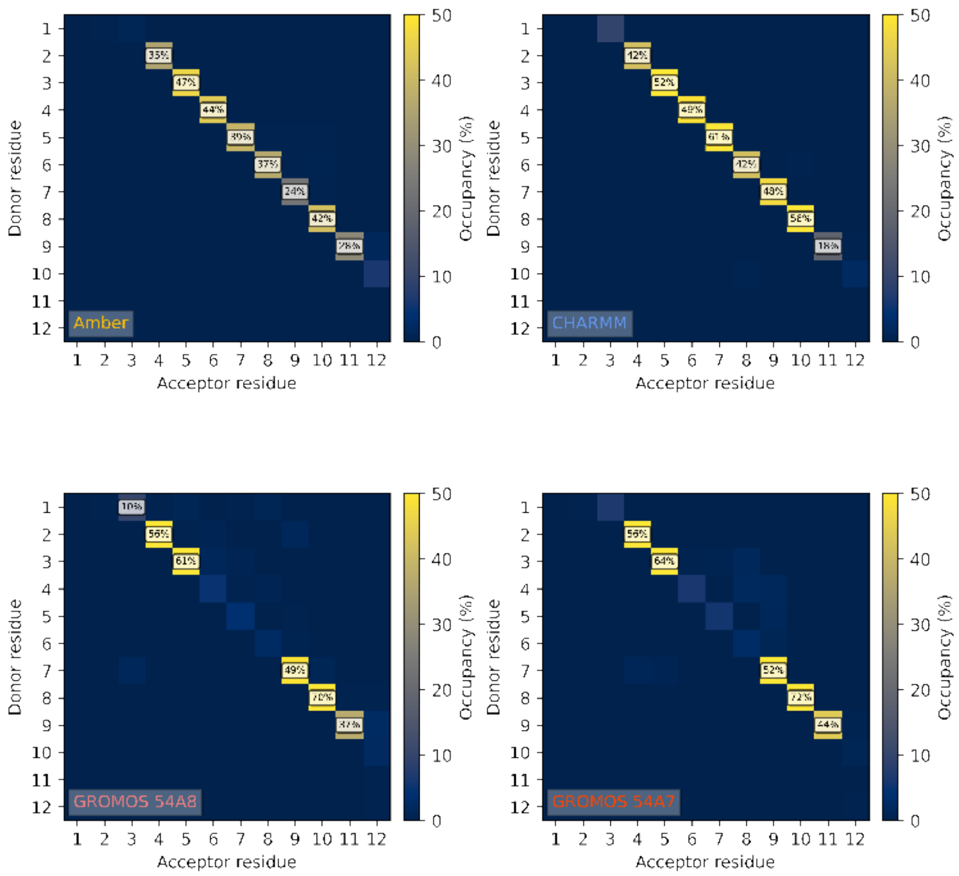

Figure S16: Intra-chain hydrogen bond occupancy map in the simulation about Unfolding of a single strand of Peptide VII in water from 3_{14} helix. Only occupancies greater than 10% are labeled.

3.17 Deaggregation of the Peptide VII octamer in water

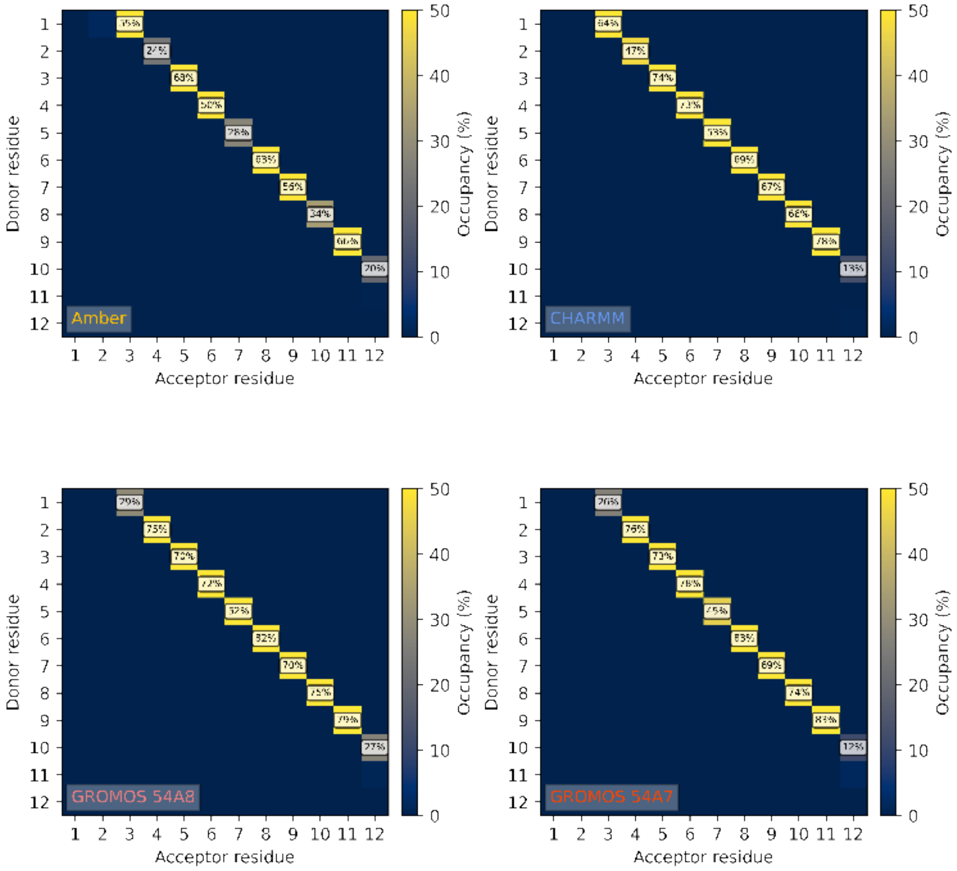

Figure S17: Intra-chain hydrogen bond occupancy map in the simulation about Deaggregation of the Peptide VII octamer in water. Only occupancies greater than 10% are labeled.

4 Time-evolution of the Helicity Score

4.1 Peptide I

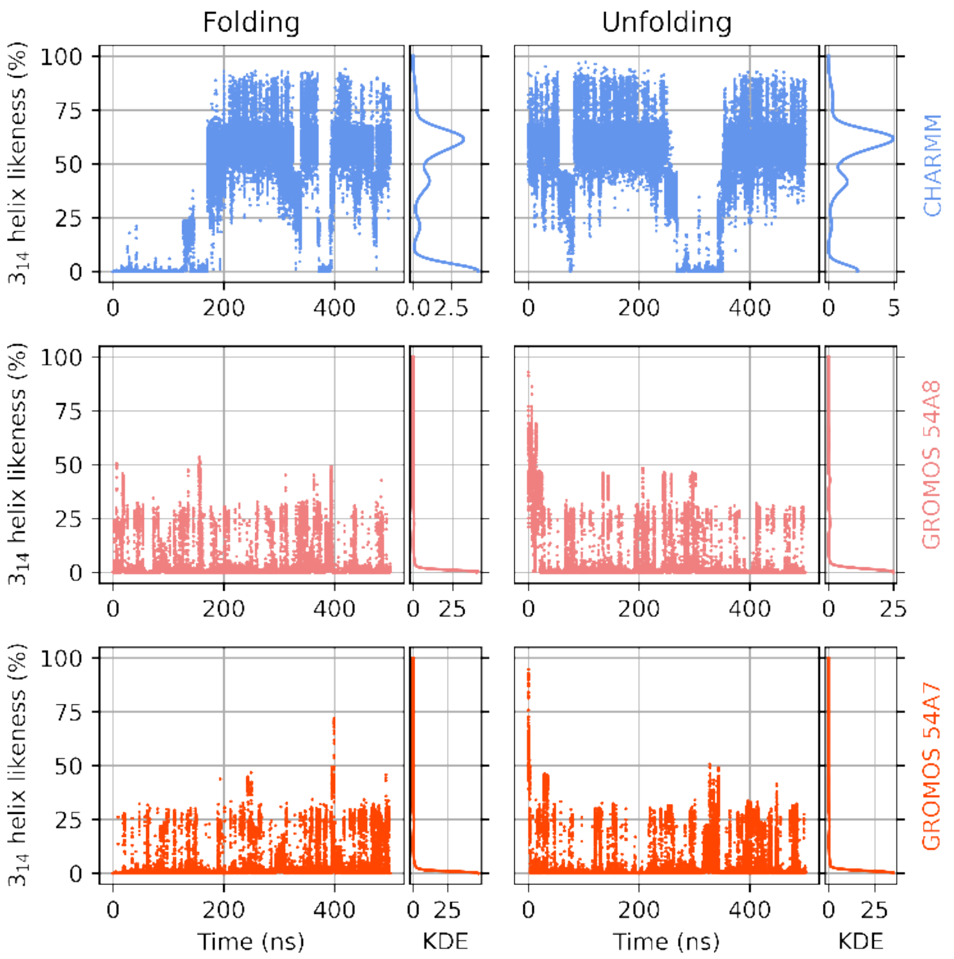

Figure S18: Time-evolution of the helicity parameter of peptide I with different force fields, started from extended (left) and helical (right) conformation. From all independent repetitions, the *best-case* results are shown, i.e. where the average helicity score is the *highest*.

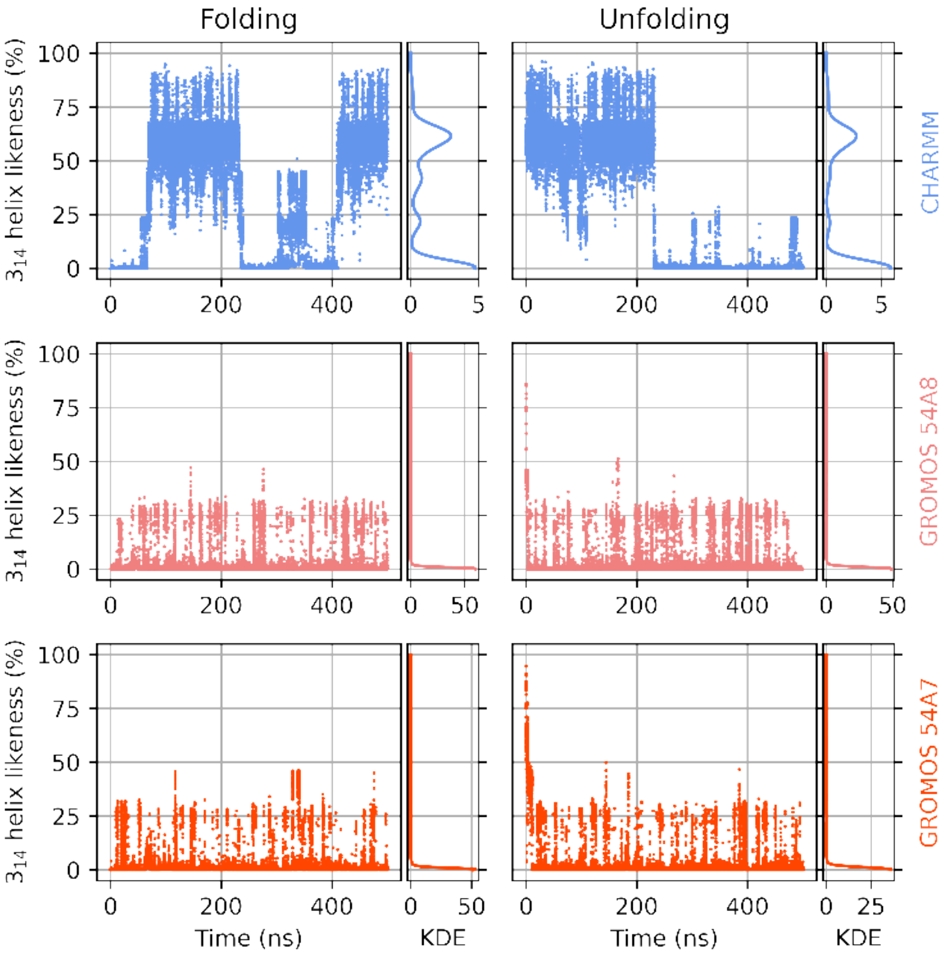

Figure S19: Time-evolution of the helicity parameter of peptide I with different force fields, started from extended (left) and helical (right) conformation. From all independent repetitions, the *worst-case* results are shown, i.e. where the average helicity score is the *lowest*.

4.2 Peptide II

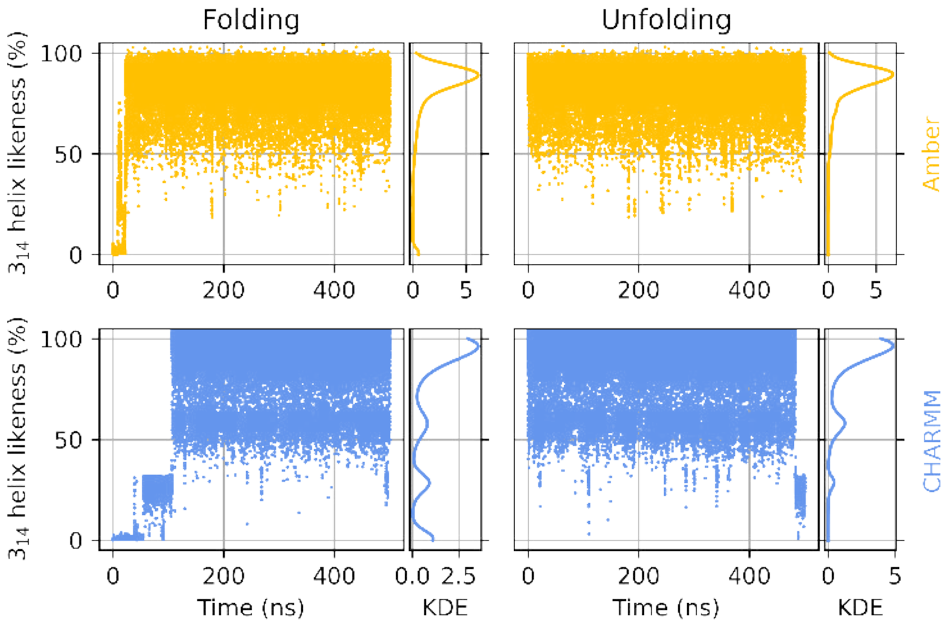

Figure S20: Time-evolution of the helicity parameter of peptide II with different force fields, started from extended (left) and helical (right) conformation. From all independent repetitions, the *best-case* results are shown, i.e. where the average helicity score is the *highest*.

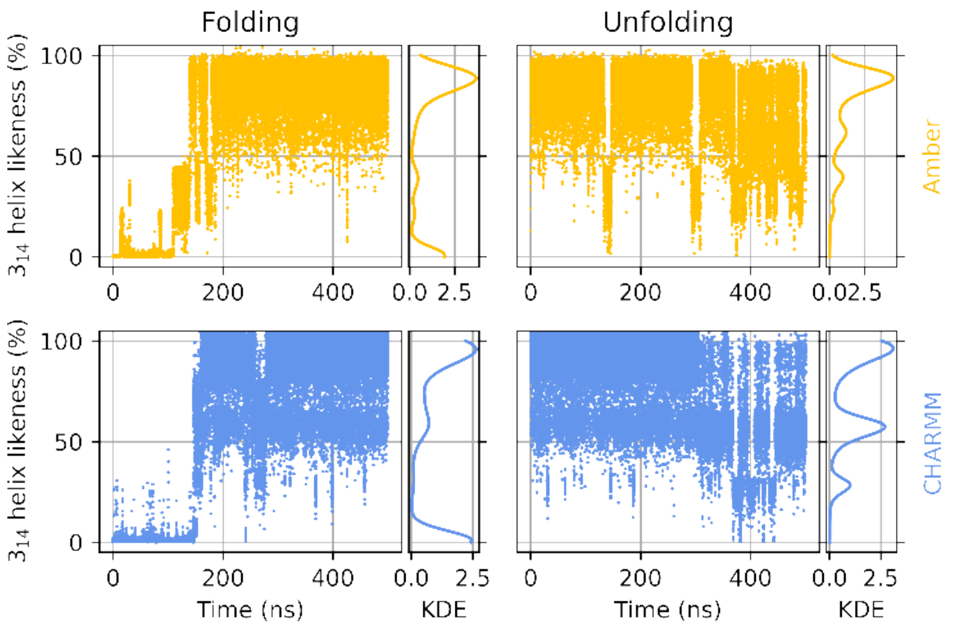

Figure S21: Time-evolution of the helicity parameter of peptide II with different force fields, started from extended (left) and helical (right) conformation. From all independent repetitions, the *worst-case* results are shown, i.e. where the average helicity score is the *lowest*.

4.3 Peptide III

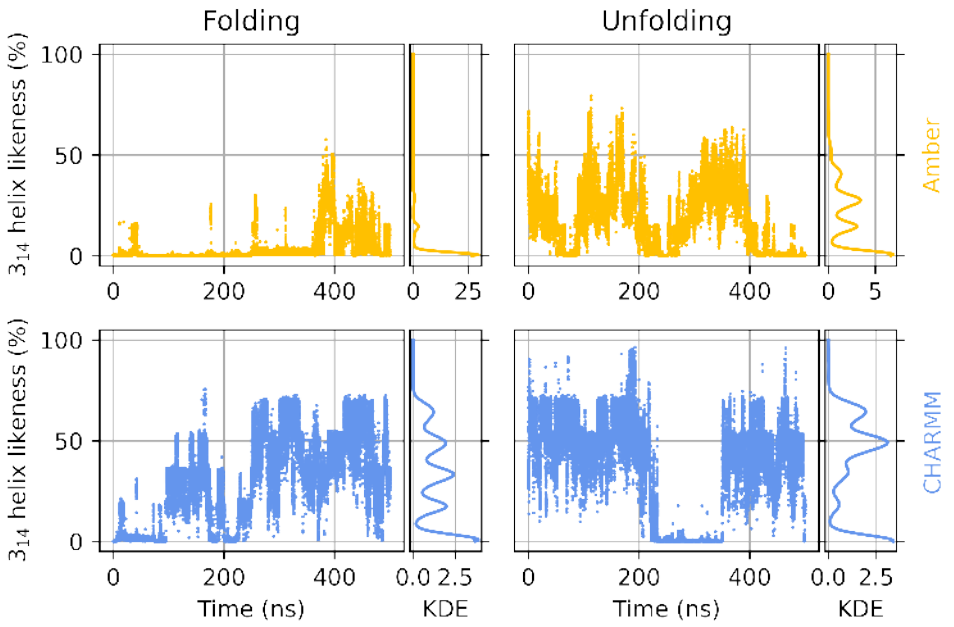

Figure S22: Time-evolution of the helicity parameter of peptide III with different force fields, started from extended (left) and helical (right) conformation. From all independent repetitions, the *best-case* results are shown, i.e. where the average helicity score is the *highest*.

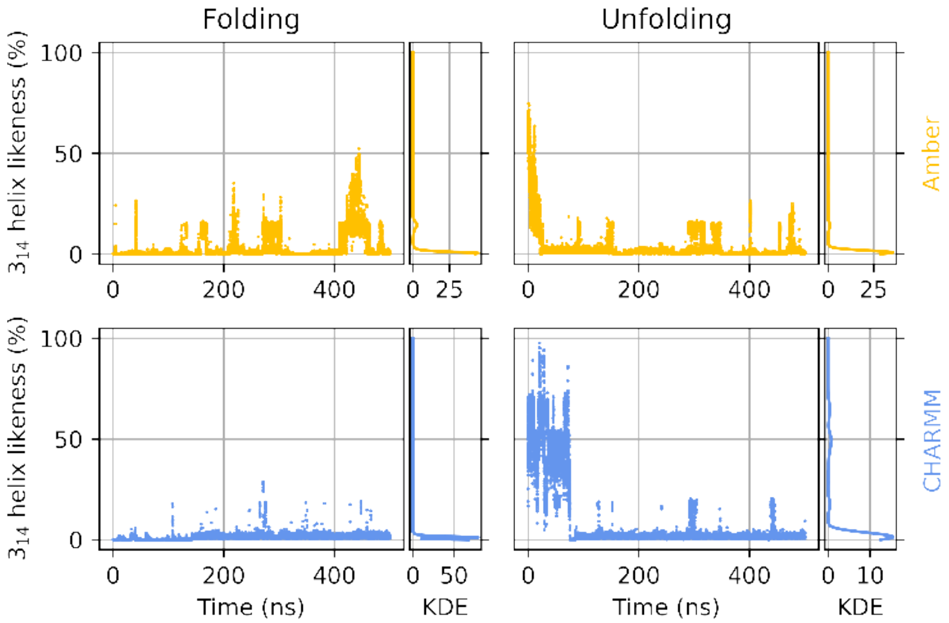

Figure S23: Time-evolution of the helicity parameter of peptide III with different force fields, started from extended (left) and helical (right) conformation. From all independent repetitions, the *worst-case* results are shown, i.e. where the average helicity score is the *lowest*.

4.4 Peptide IV

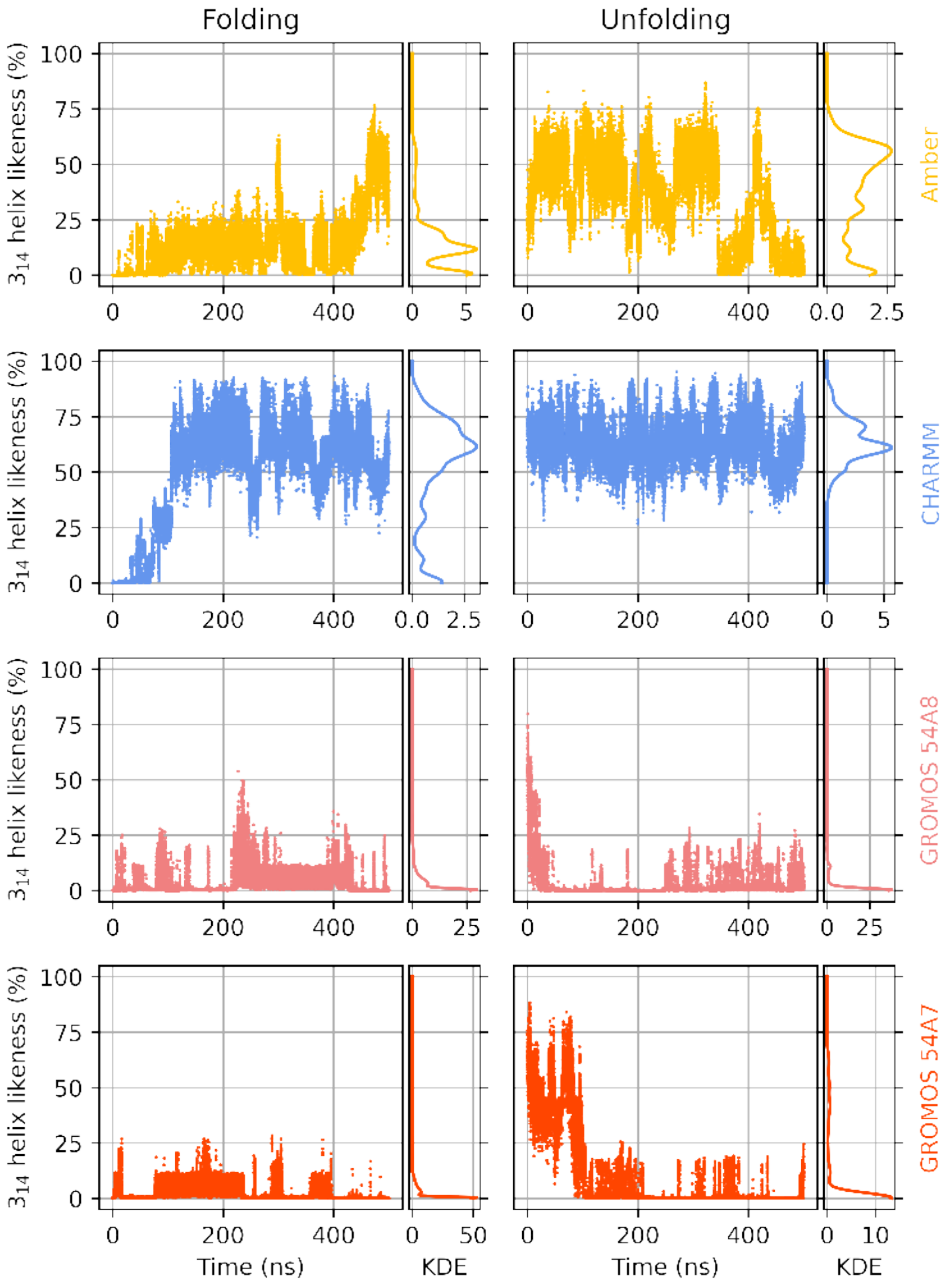

Figure S24: Time-evolution of the helicity parameter of peptide IV with different force fields, started from extended (left) and helical (right) conformation. From all independent repetitions, the *best-case* results are shown, i.e. where the average helicity score is the *highest*.

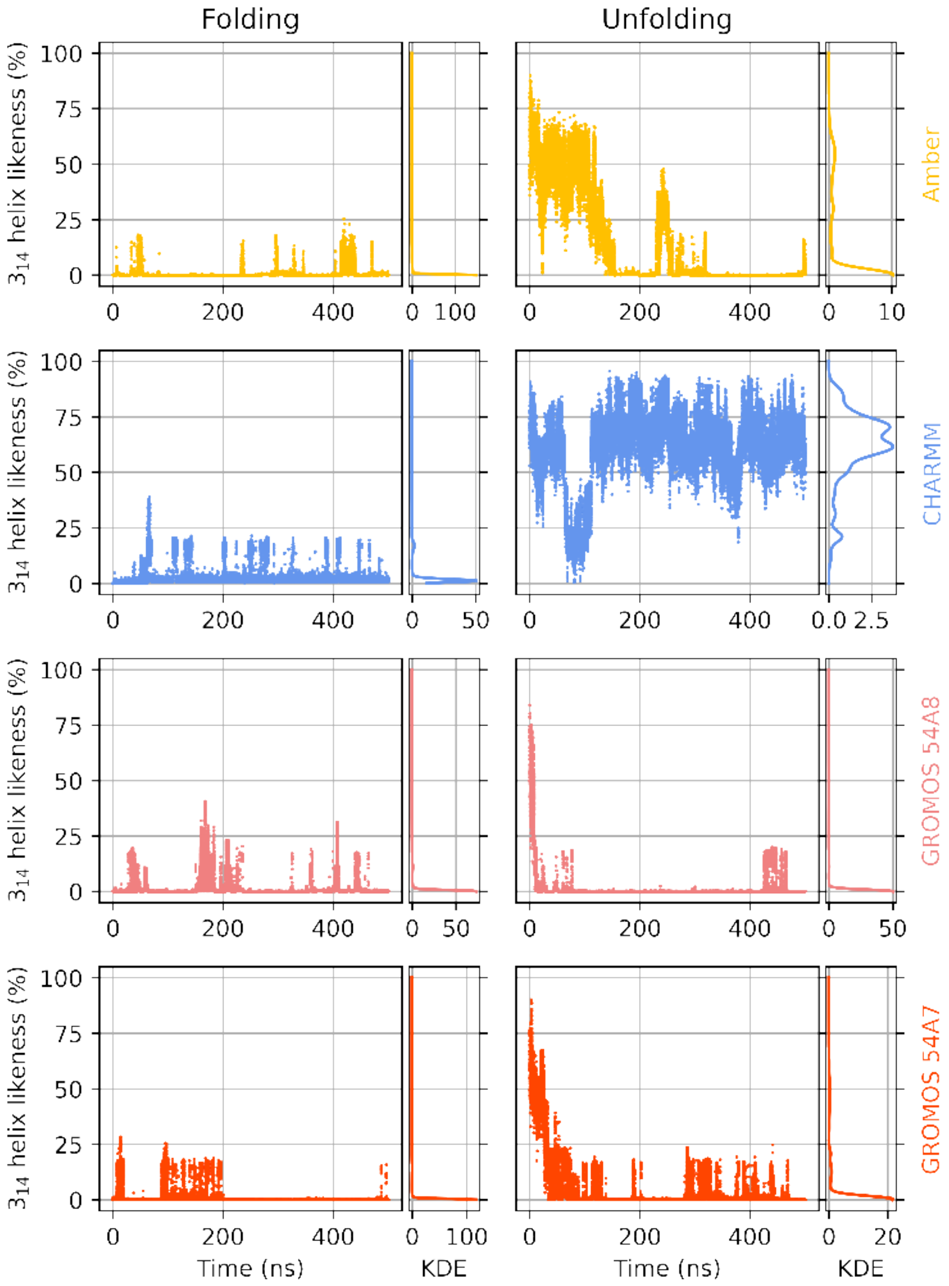

Figure S25: Time-evolution of the helicity parameter of peptide IV with different force fields, started from extended (left) and helical (right) conformation. From all independent repetitions, the *worst-case* results are shown, i.e. where the average helicity score is the *lowest*.

5 Time-evolution of the Hairpin Conformation Likeness Score

5.1 Peptide V

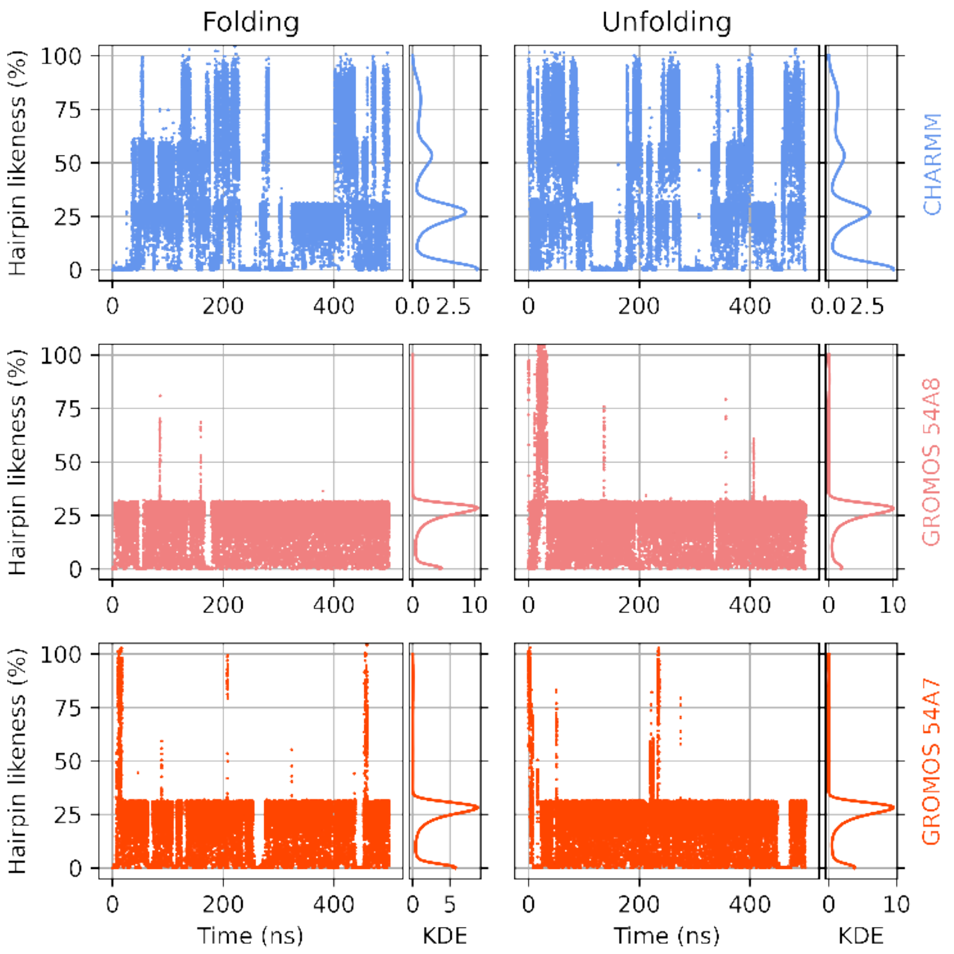

Figure S26: Time-evolution of the hairpin conformational likeness parameter of peptide V with different force fields, started from extended (left) and hairpin (right) conformation. From all independent repetitions, the *best-case* results are shown, i.e. where the average hairpinity score is the *highest*.

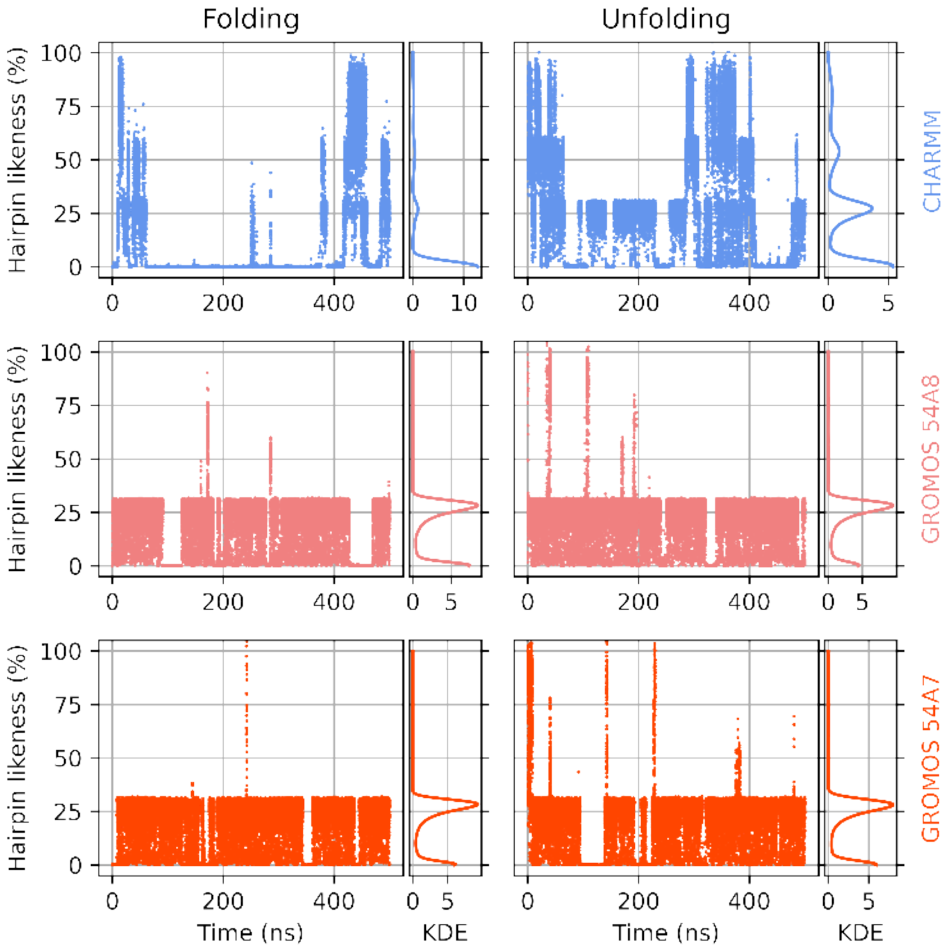

Figure S27: Time-evolution of the hairpin conformational likeness parameter of peptide V with different force fields, started from extended (left) and hairpin (right) conformation. From all independent repetitions, the *worst-case* results are shown, i.e. where the average hairpinity score is the *lowest*.

6 Root Mean Square Deviation of Peptide VII from the Reference Structure

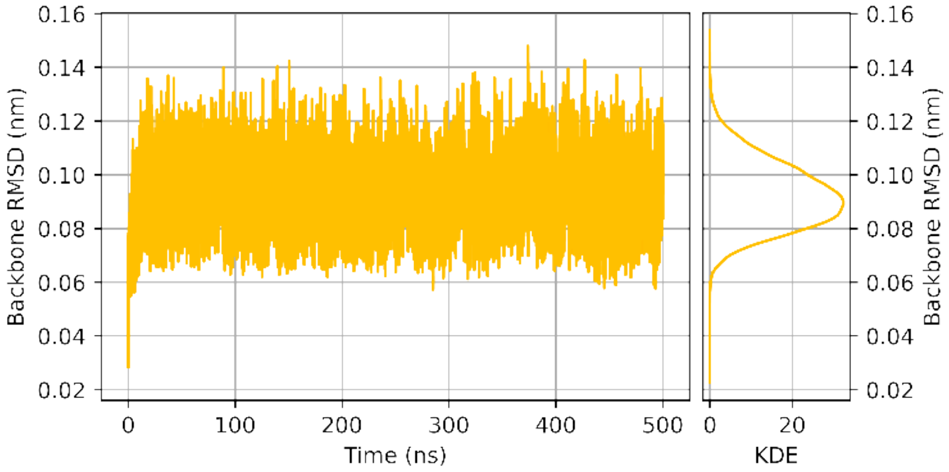

Figure S28: Time-dependence of the root mean square deviation from the reference structure of peptide VII, by force field amber

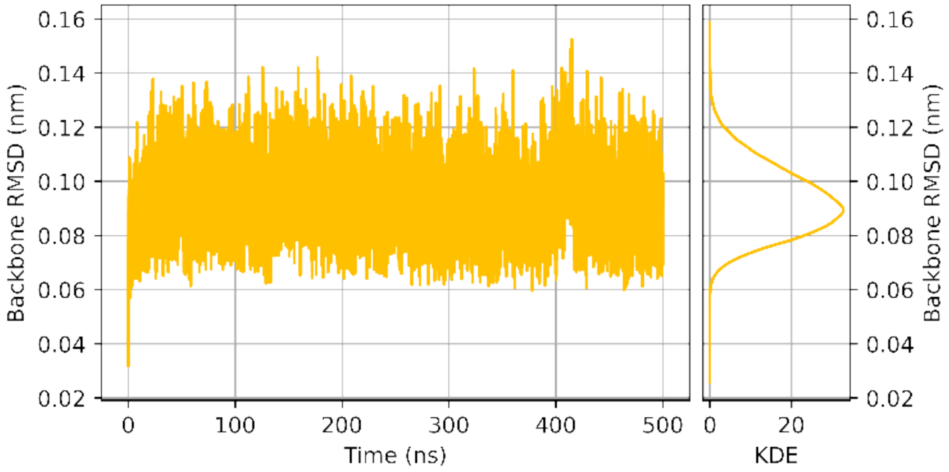

Figure S29: Time-dependence of the root mean square deviation from the reference structure of peptide VII, by force field amber

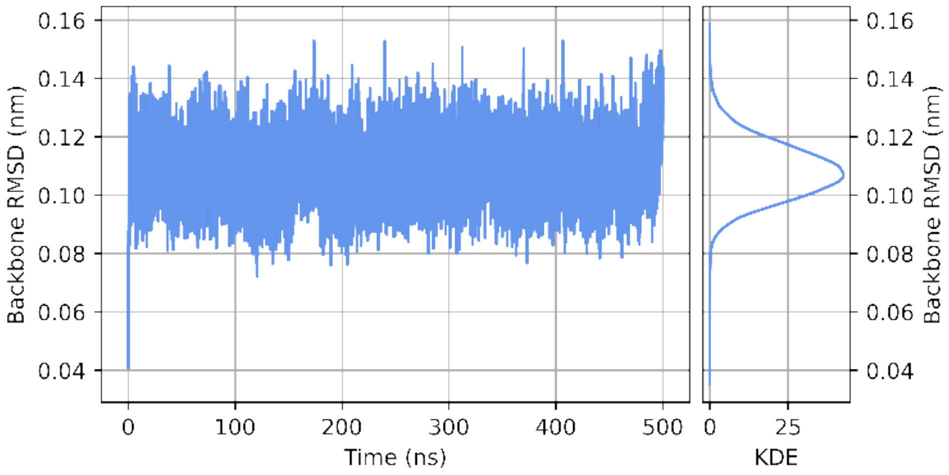

Figure S30: Time-dependence of the root mean square deviation from the reference structure of peptide VII, by force field charmm

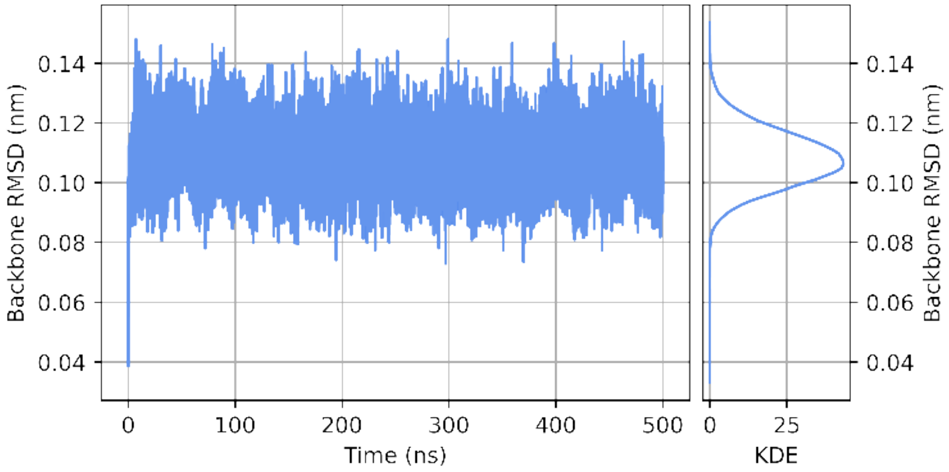

Figure S31: Time-dependence of the root mean square deviation from the reference structure of peptide VII, by force field charmm

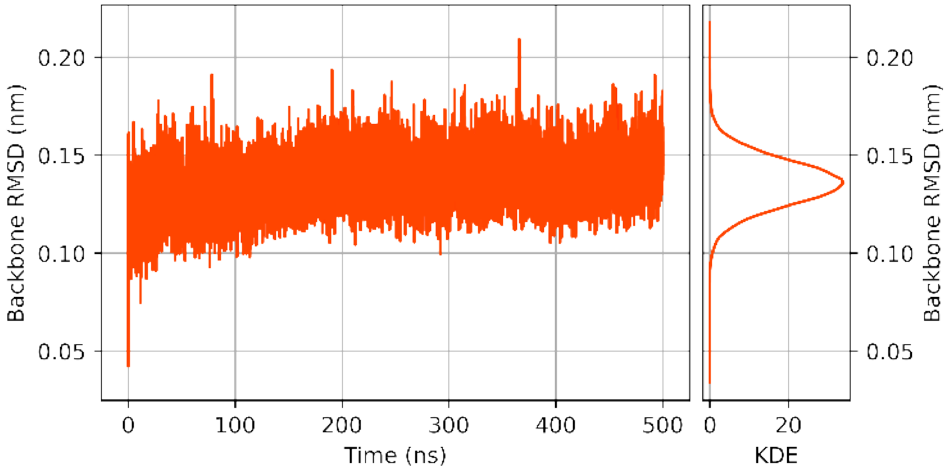

Figure S32: Time-dependence of the root mean square deviation from the reference structure of peptide VII, by force field gromos54a7

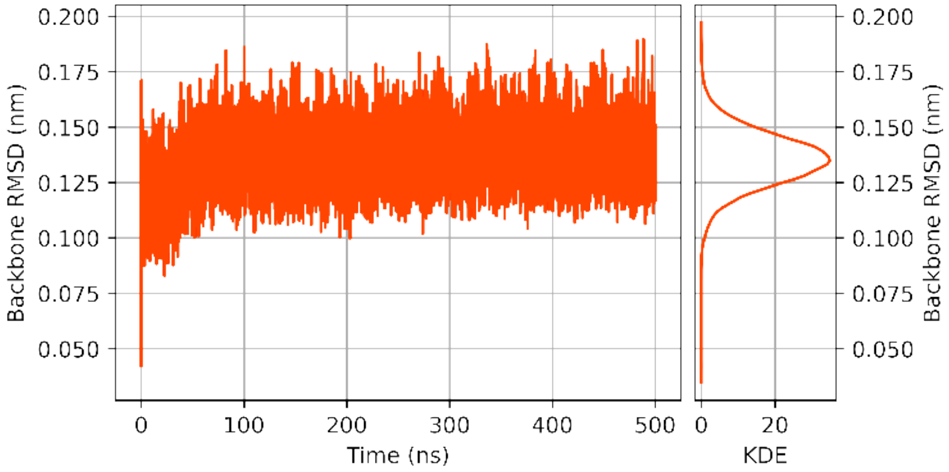

Figure S33: Time-dependence of the root mean square deviation from the reference structure of peptide VII, by force field gromos54a7

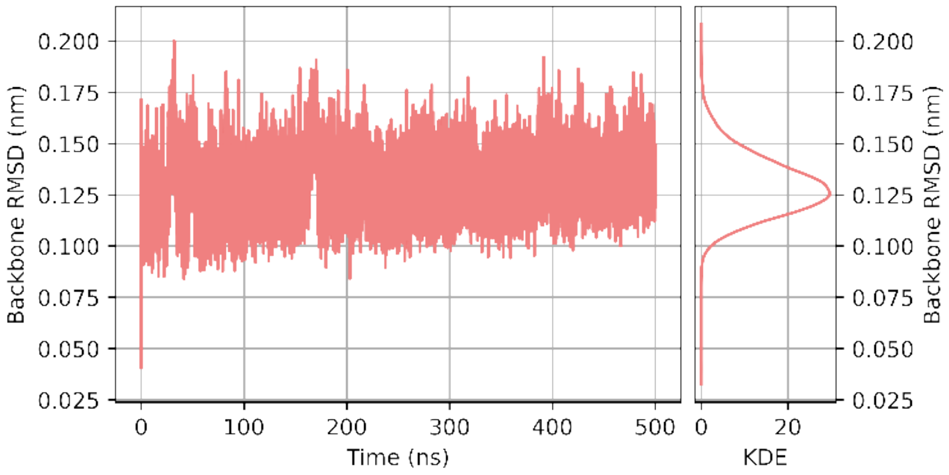

Figure S34: Time-dependence of the root mean square deviation from the reference structure of peptide VII, by force field gromos

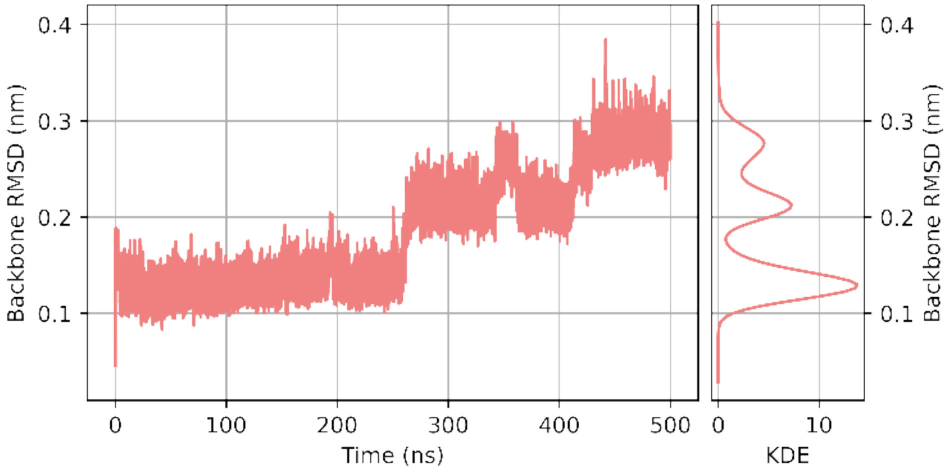

Figure S35: Time-dependence of the root mean square deviation from the reference structure of peptide VII, by force field gromos

7 NOE Distance Limit Violations

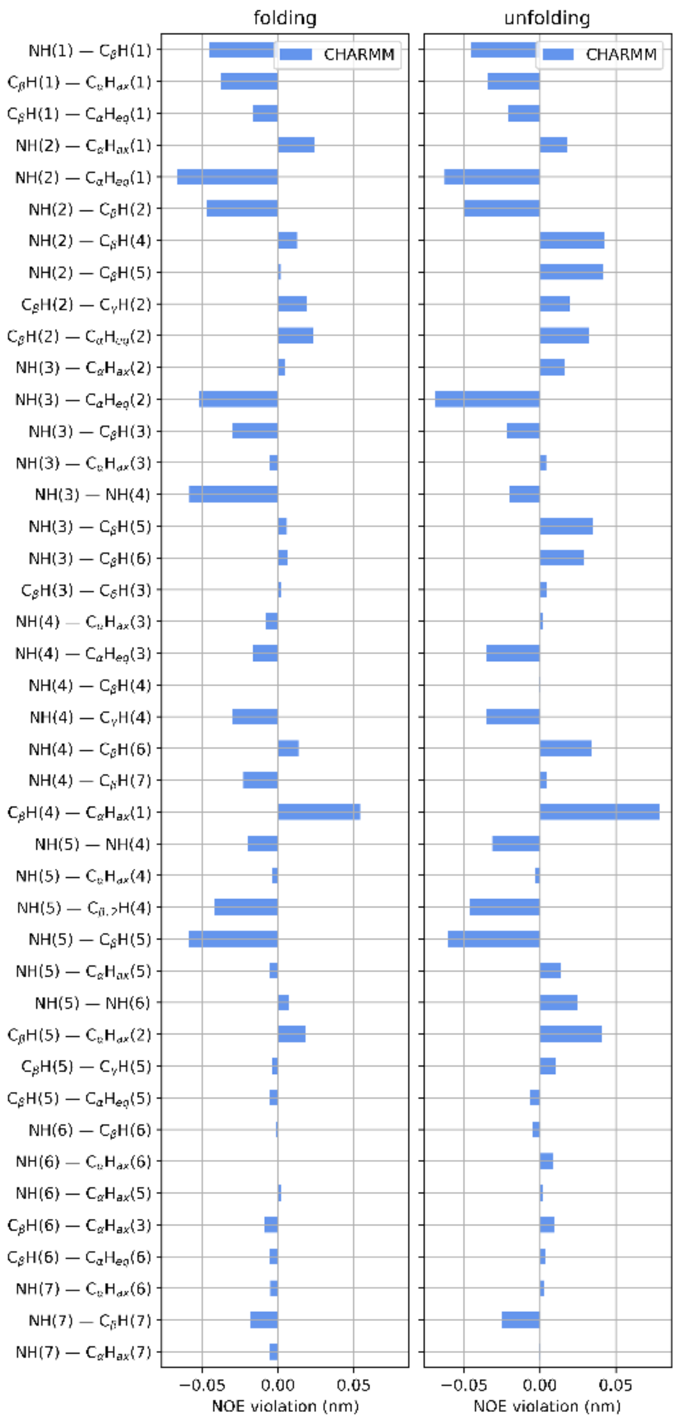

Figure S36: Upper NOE distance limit violations in simulations of peptide I

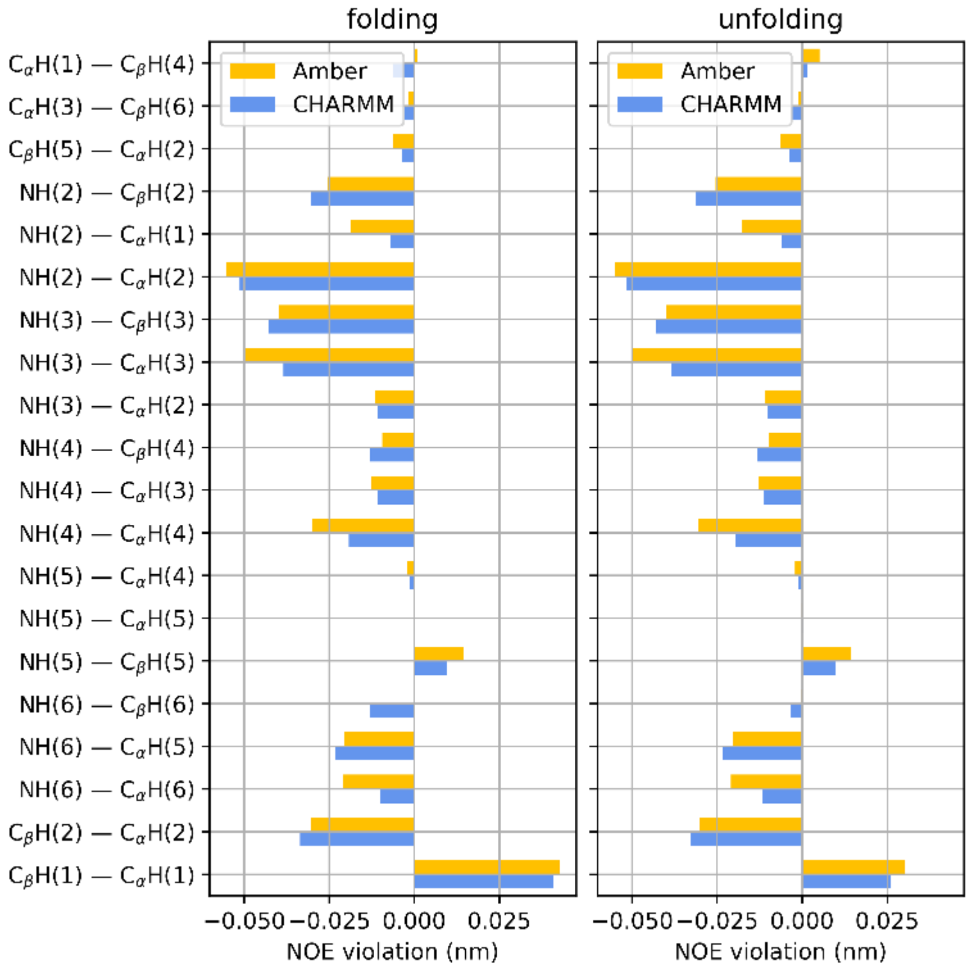

Figure S37: Upper NOE distance limit violations in simulations of peptide II

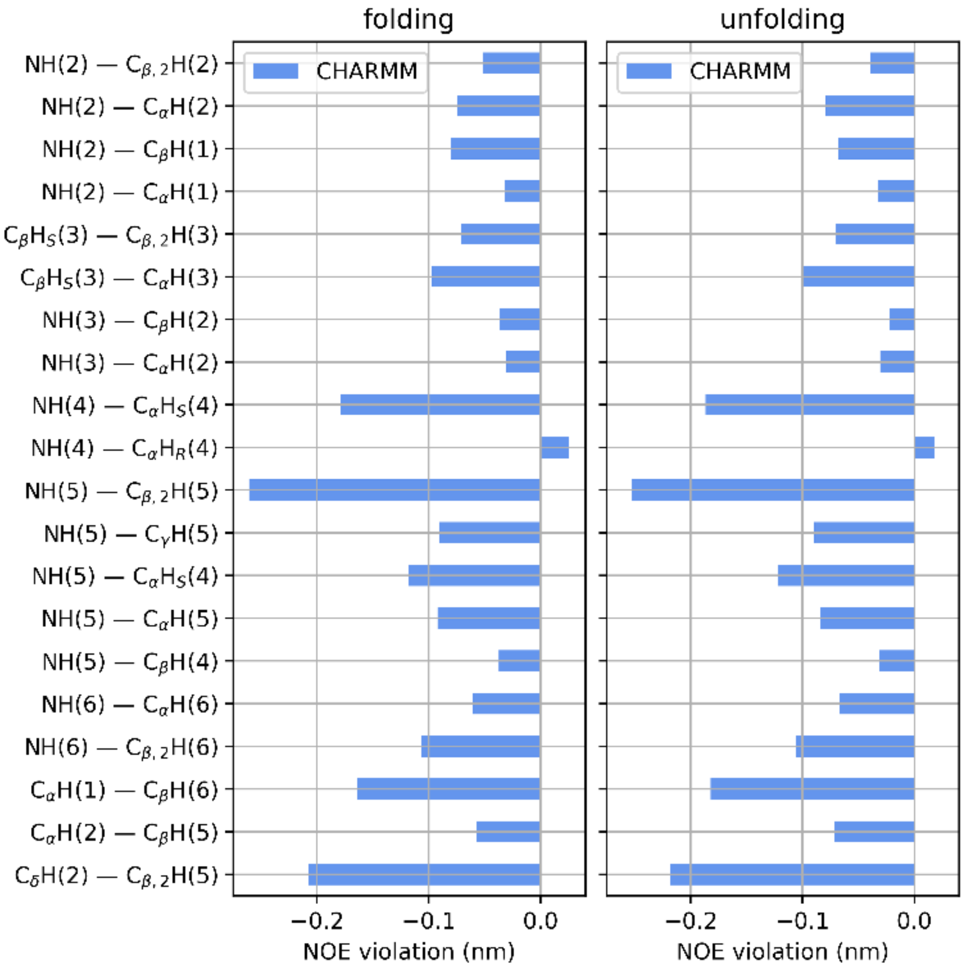

Figure S38: Upper NOE distance limit violations in simulations of peptide V

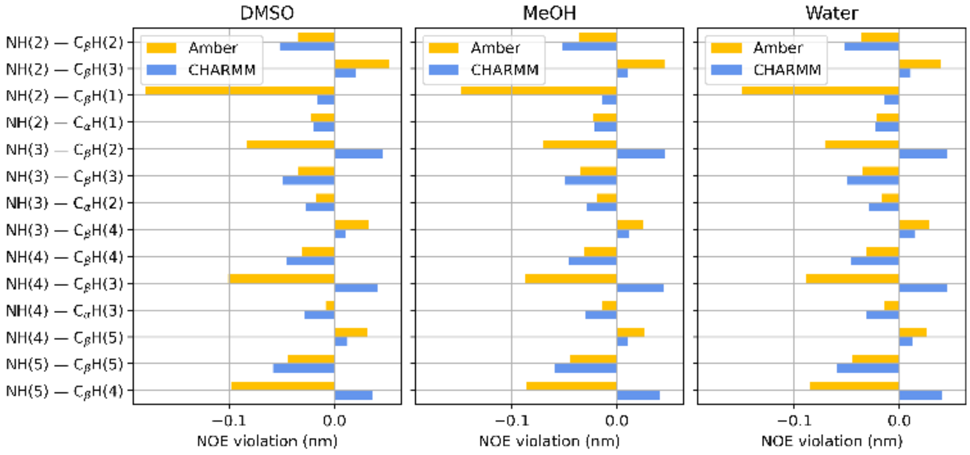

Figure S39: Upper NOE distance limit violations in simulations of peptide VI

8 Cumulative cluster counts

8.1 Peptide I

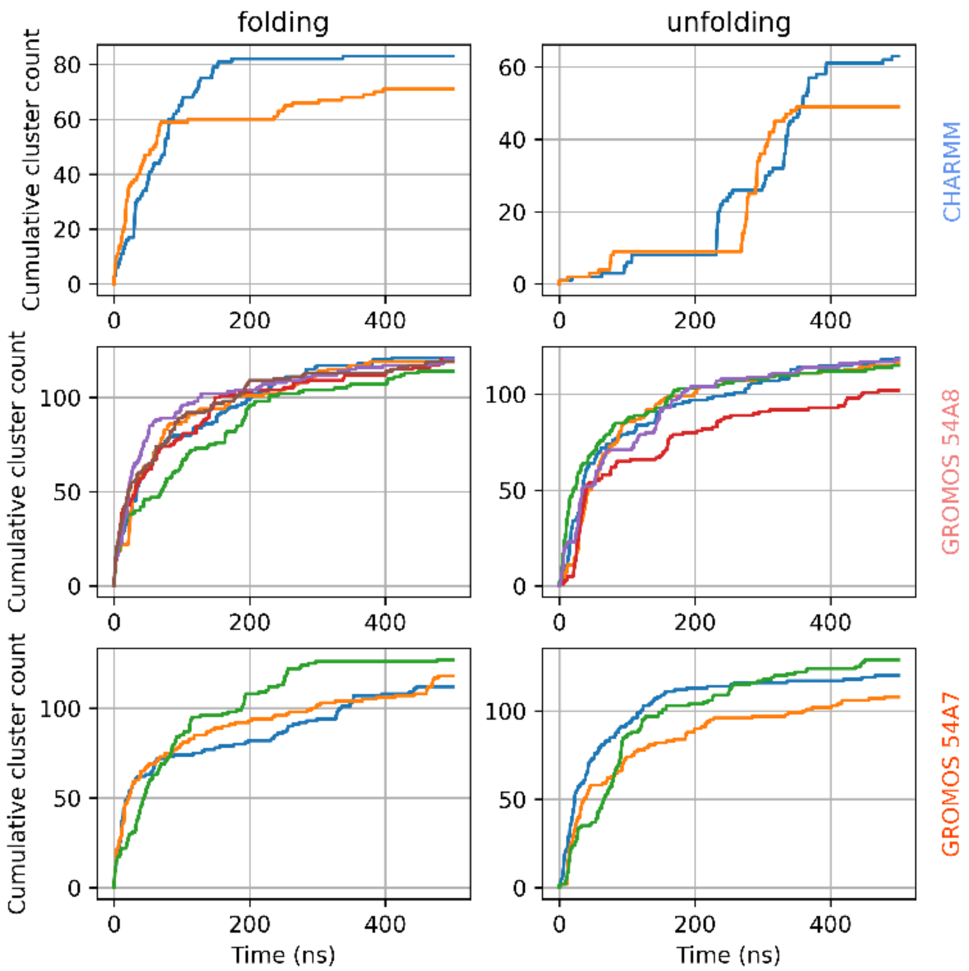

Figure S40: Cumulative cluster count in simulations of peptide I

8.2 Peptide II

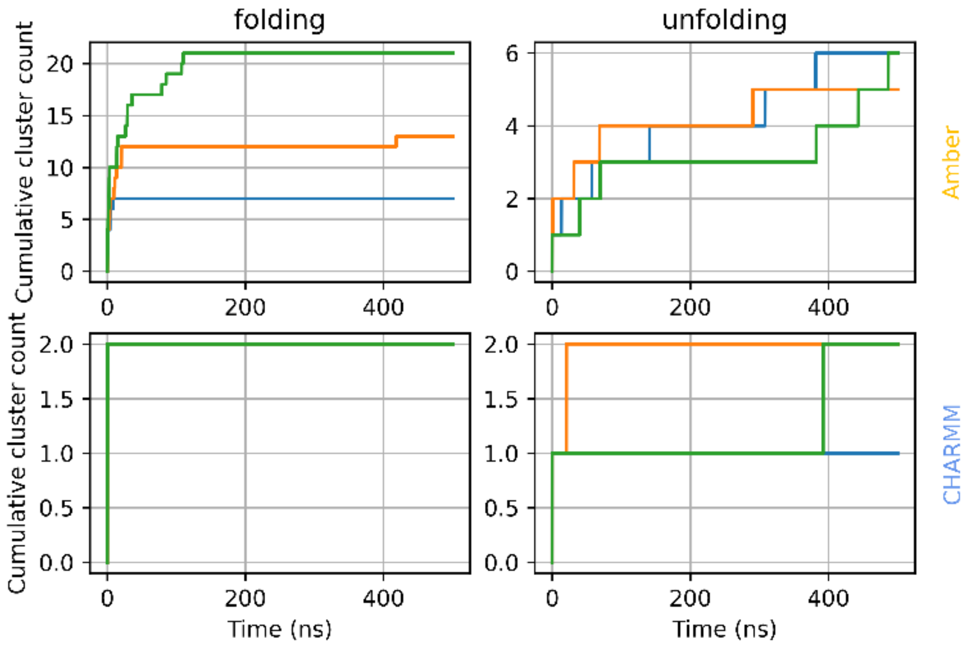

Figure S41: Cumulative cluster count in simulations of peptide II

8.3 Peptide III

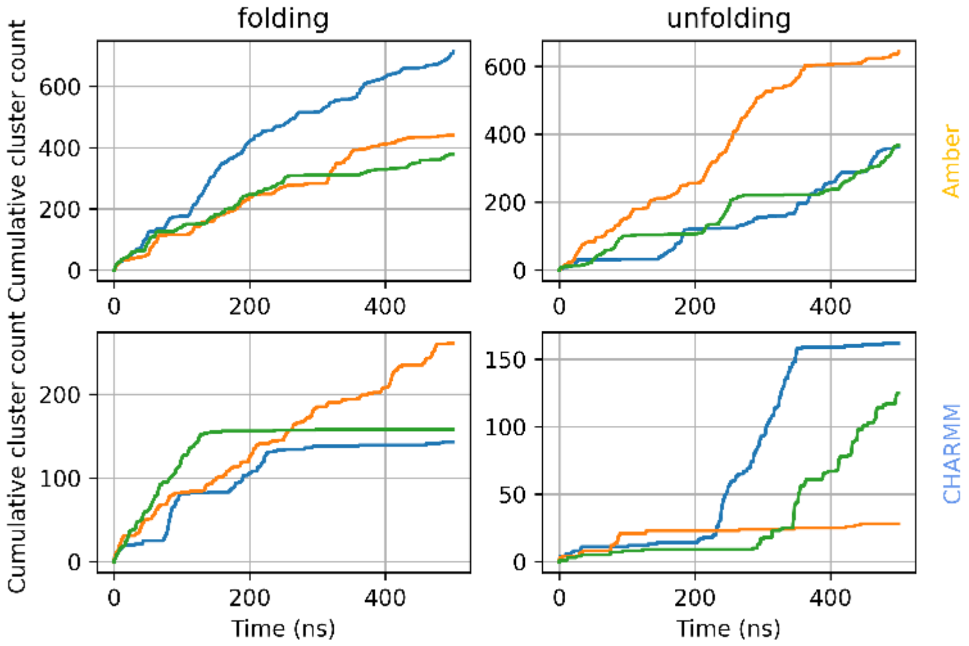

Figure S42: Cumulative cluster count in simulations of peptide III

8.4 Peptide IV

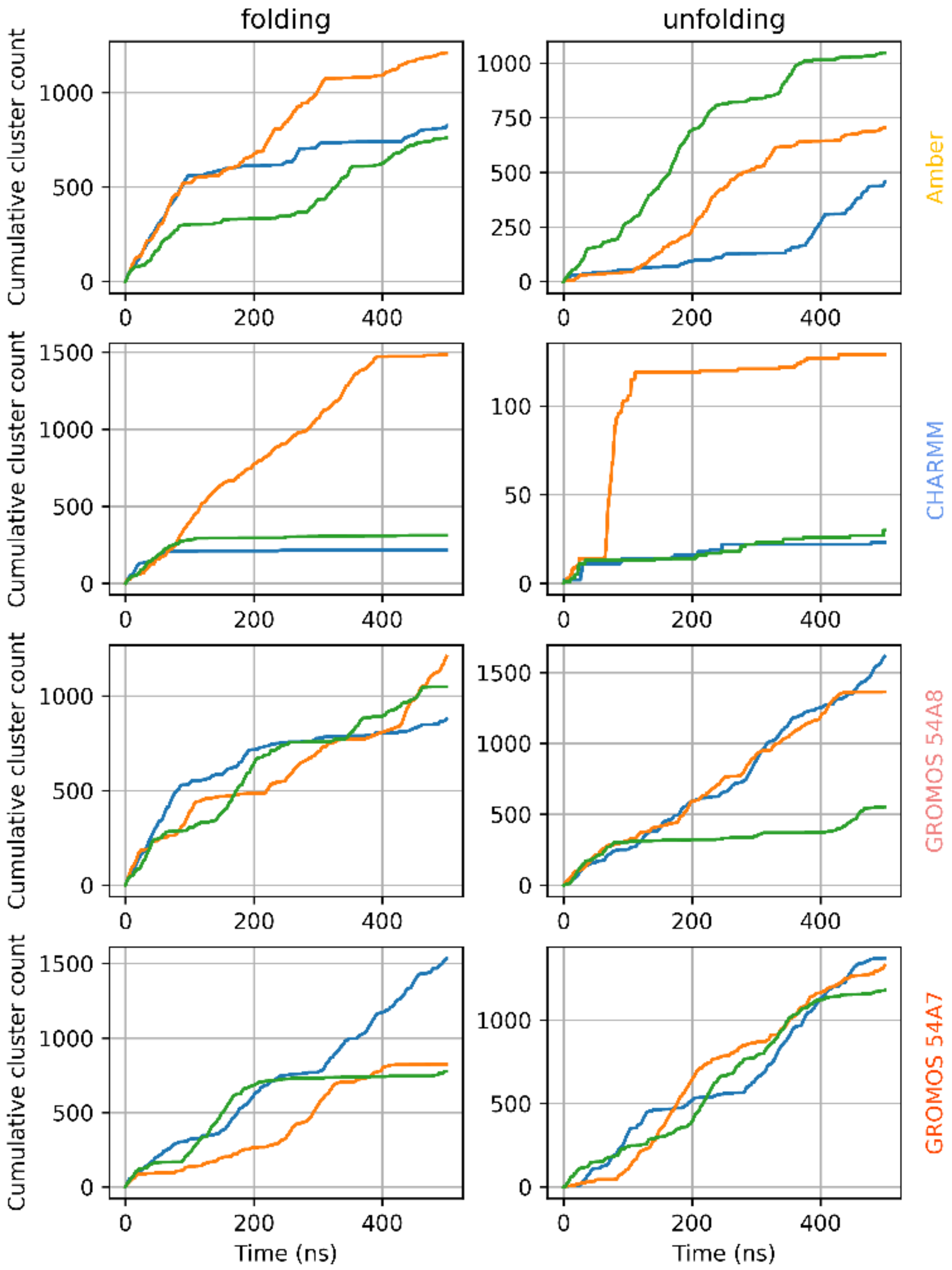

Figure S43: Cumulative cluster count in simulations of peptide IV

8.5 Peptide V

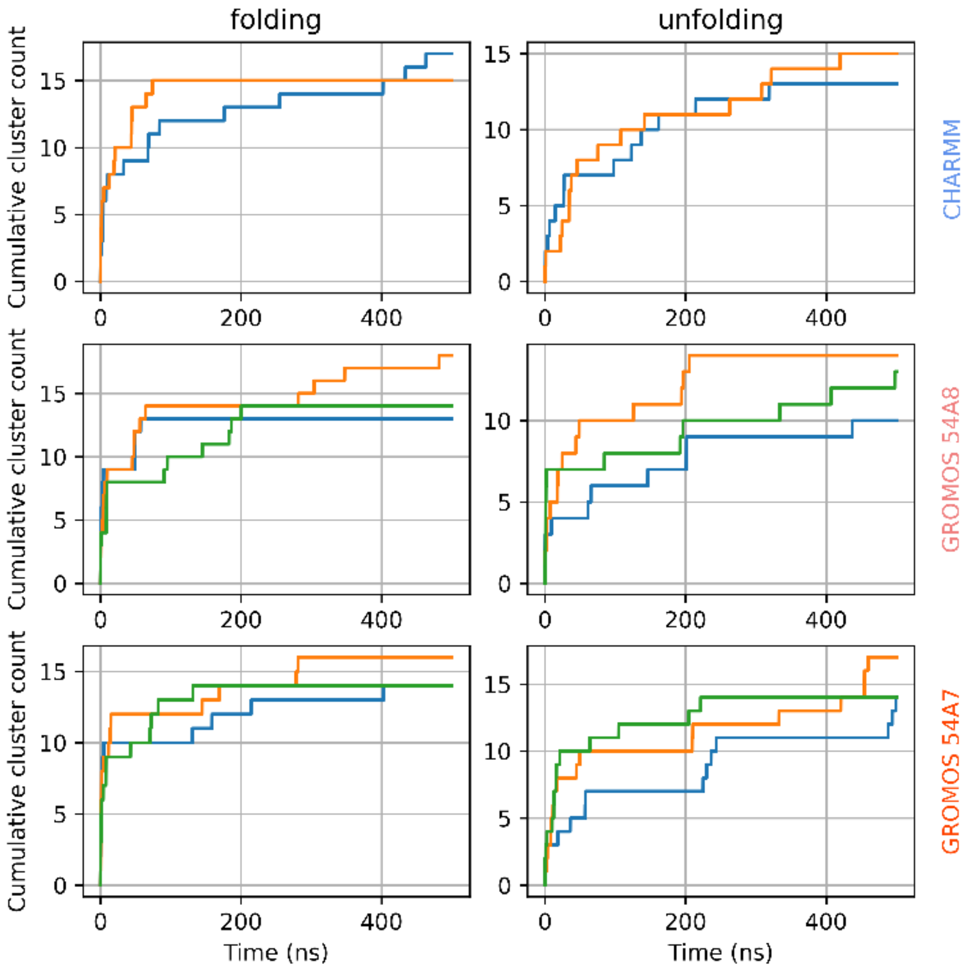

Figure S44: Cumulative cluster count in simulations of peptide V

8.6 Peptide VI

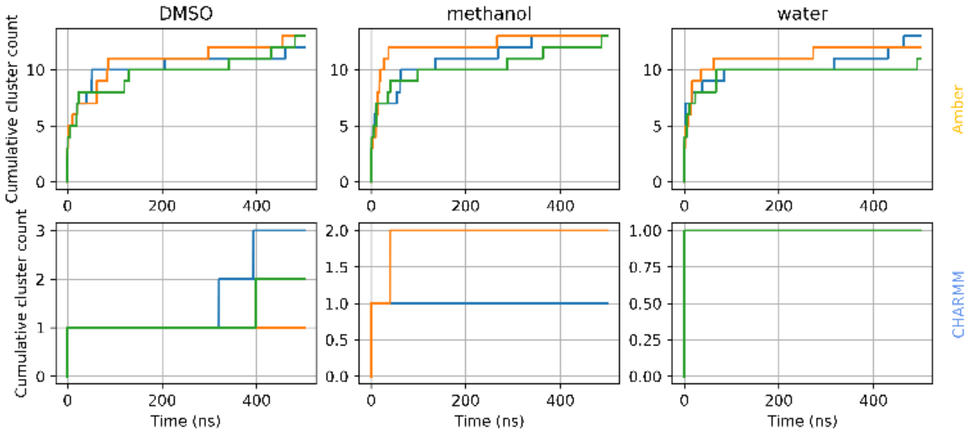

Figure S45: Cumulative cluster count in simulations of peptide VI

8.7 Peptide VII

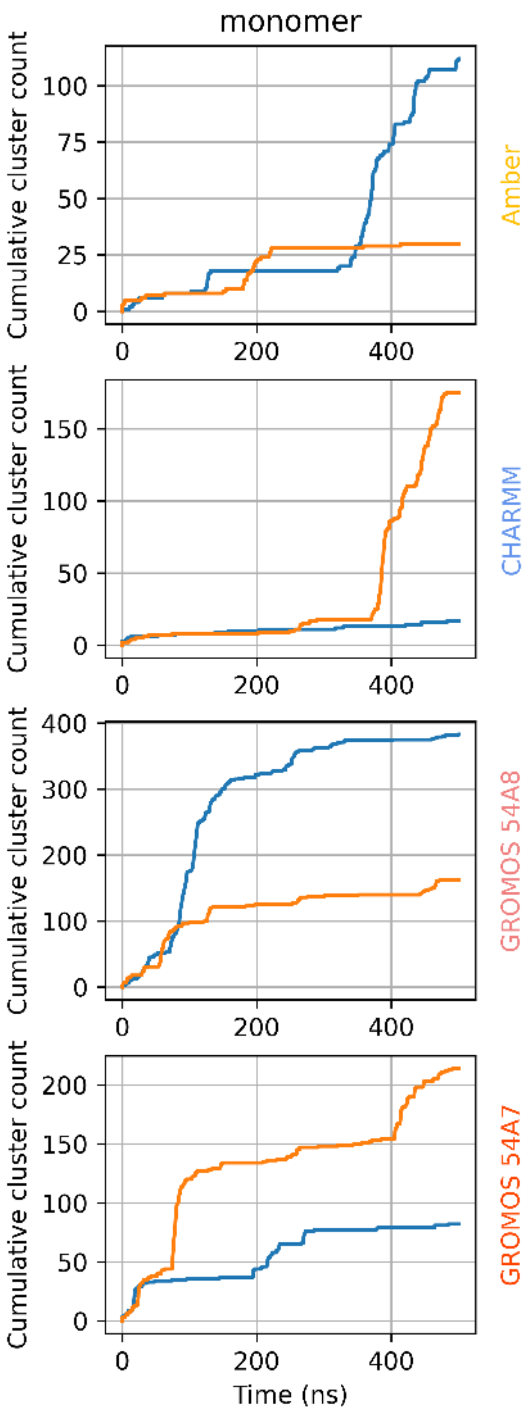

Figure S46: Cumulative cluster count in simulations of peptide VII
